# Calcium-dependent protein kinases responsible for the phosphorylation of a bZIP transcription factor FD crucial for the florigen complex formation

**DOI:** 10.1038/srep08341

**Published:** 2015-02-09

**Authors:** Nozomi Kawamoto, Michiko Sasabe, Motomu Endo, Yasunori Machida, Takashi Araki

**Affiliations:** 1 Division of Integrated Life Science, Graduate School of Biostudies, Kyoto University, Yoshida-Konoe-cho, Sakyo-ku, Kyoto, 606-8501, Japan; 2 Division of Biological Science, Graduate School of Science, Nagoya University, Furo-cho, Chikusa-ku, Nagoya, 464-8602, Japan

## Abstract

Appropriate timing of flowering is critical for reproductive success and necessarily involves complex genetic regulatory networks. A mobile floral signal, called florigen, is a key molecule in this process, and FLOWERING LOCUS T (FT) protein is its major component in *Arabidopsis.* FT is produced in leaves, but promotes the floral transition in the shoot apex, where it forms a complex with a basic region/leucine-zipper (bZIP) transcription factor, FD. Formation of the florigen complex depends on the supposed phosphorylation of FD; hitherto, however, the responsible protein kinase(s) have not been identified. In this study, we prepared protein extracts from shoot apices of plants around the floral transition, and detected a protein kinase activity that phosphorylates a threonine residue at position 282 of FD (FD T282), which is a crucial residue for the complex formation with FT via 14-3-3. The kinase activity was calcium-dependent. Subsequent biochemical, cellular, and genetic analyses showed that three calcium-dependent protein kinases (CDPKs) efficiently phosphorylate FD T282. Two of them (CPK6 and CPK33) are expressed in shoot apical meristem and directly interact with FD, suggesting they have redundant functions. The loss of function of one CDPK (CPK33) resulted in a weak but significant late-flowering phenotype.

Plants decide the appropriate time to flower by sensing environmental cues including photoperiod, light quality, and ambient temperature, as well as endogenous cues. Adaptive seasonal flowering is an important trait that ensures successful sexual reproduction. Genetic and molecular analyses in *Arabidopsis* and other plants showed that complex regulatory networks consisting of multiple pathways contribute to the regulation of vegetative-to-reproductive phase transition (floral transition) via transcriptional regulation of several key genes including *FLOWERING LOCUS T* (*FT*)^1^,^2^. *FT* encodes a 20 kDa protein with homology to the mammalian phosphatidylethanolamine binding protein (PEBP) family^3^,^4^ and acts as a mobile flowering signal, florigen^5^,^6^,^7^,^8^,^9^,^10^,^11^,^12^. FT is transcribed and translated in the phloem companion cells of the leaf, and moves to the shoot apical meristem through the phloem^10^,^13^. In the shoot apical meristem, FT forms a complex with a basic region/leucine-zipper (bZIP) transcription factor FD^5^,^6^. As mentioned below, this complex formation is likely to be mediated by 14-3-3 proteins. The FT-FD florigen complex promotes floral transition by transcriptional activation of floral meristem identity genes, including *APETALA1* (*AP1*), that in turn orchestrate flower development via transcriptional regulation of genes involved in organ identity specification^5^,^6^,^14^. While FD does not directly bind to the promoter of *AP1*^15^, FD is a key factor that links florigen FT and initiation of floral development^5^,^6^.

As is the case for FT, the role of FD in the regulation of flowering is widely conserved^16^,^17^,^18^,^19^,^20^,^21^. FT and FD are interdependent partners through protein-protein interactions^5^,^6^. A previous study showed that a threonine residue at position 282 (T282) of FD, which is a potential phosphorylation site for calcium-dependent protein kinases (CDPKs) or Snf1-related protein kinases (SnRKs), is crucial for the FD-FT complex formation and function, as assayed by complementation of *fd-1*^5^. In rice, interaction of HEADING DATE 3a (a rice counterpart of FT) and OsFD1 is not a direct one, but is mediated by 14-3-3, a phospho-protein binding protein^19^. In Arabidopsis, it was shown that certain isoforms of 14-3-3 interact with FT^22^,^23^. Thus, it has been assumed that FD is phosphorylated by unidentified protein kinases, and 14-3-3 proteins recognize and interact with the phosphorylated FD as an adaptor protein to form the FD-FT complex. Of six PEBPs in Arabidopsis (FT, TWIN SISTER OF FT (TSF), TERMINAL FLOWER 1 (TFL1), *Arabidopsis thaliana* CENTRORADIALIS homologue (ATC), BROTHER OF FT AND TFL1 (BFT), and MOTHER OF FT AND TFL1 (MFT)^4^), five are known to act as floral regulators. FT and TSF are floral promoters^3^,^4^,^24^, while TFL1, BFT, and ATC are repressors^25^,^26^,^27^,^28^. These PEBPs have conserved 14-3-3 binding motifs and also interact with FD^5^,^6^,^25^,^26^,^27^,^28^,^29^. Thus, phosphorylation of FD is a key step in the complex formation with FT or other PEBSs to regulate floral transition. However, key regulators of FD function, such as the protein kinases responsible for phosphorylation of T282, remain unknown. Identification of these protein kinases (hereafter referred to as FD kinases) is essential for understanding the regulation of floral transition by FD and FT in the shoot apex^30^.

In this study, we characterized kinase activities in protein extracts from shoot apices, using purified full-length FD and showed that FD kinases belong to a family of CDPKs. Further biochemical, cellular, and genetic analyses showed that 3 CDPKs (CPK4, CPK6, and CPK33; CDPK gene/protein names are according to the nomenclature in ref. 31) efficiently phosphorylate FD T282. CPK6 and CPK33 are expressed in shoot apical meristem and directly interact with FD. The loss of CPK33 function results in a weak but significant late-flowering phenotype. These results indicate that CDPKs, including CPK6 and CPK33, are responsible for the phosphorylation of FD, which is crucial for the florigen complex formation.

## Results

### Phosphorylation of FD and its requirement for interaction with FT and 14-3-3

**Figure 1:**
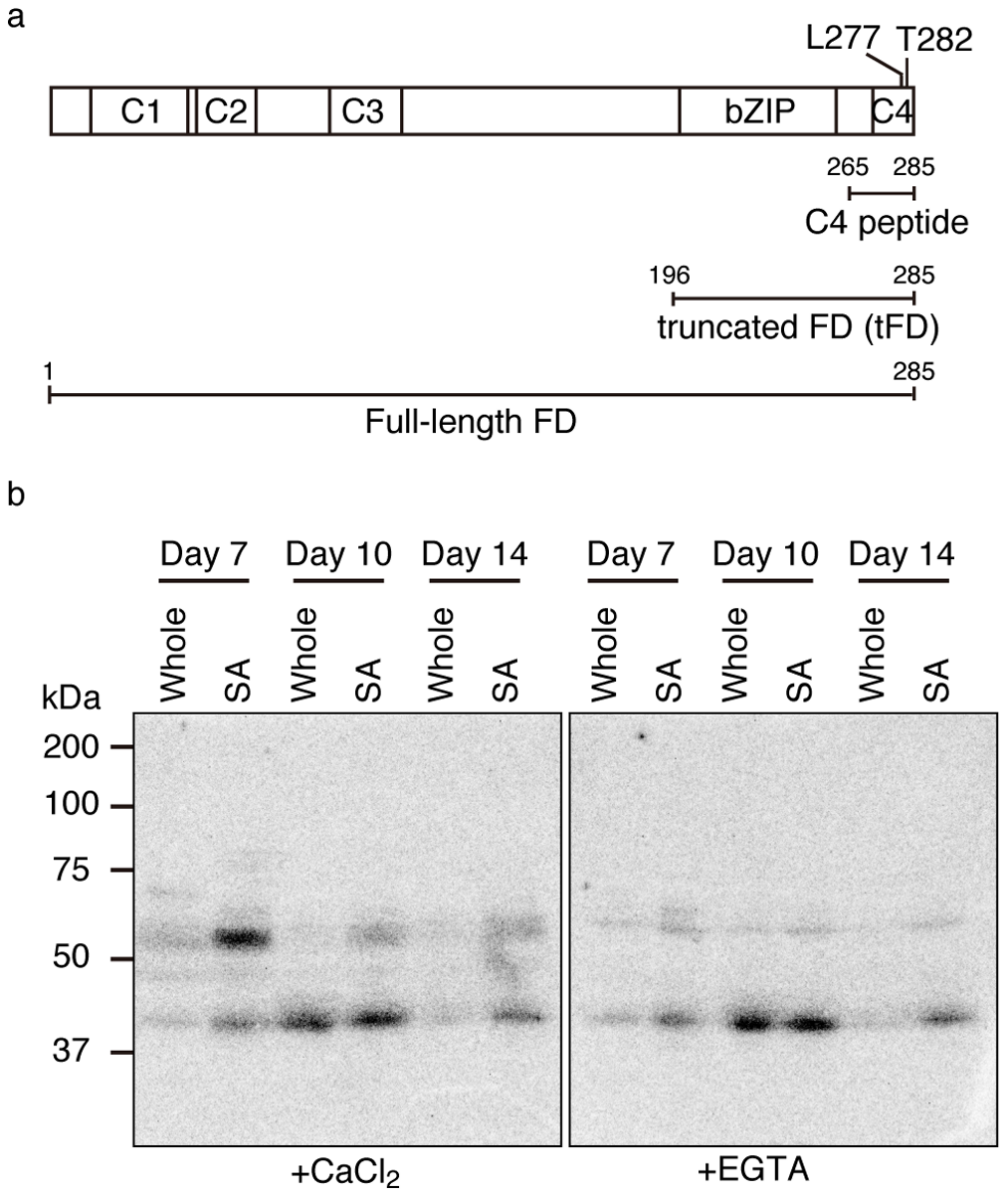
Phosphorylation of FD by kinase activity in protein extracts from shoot apices. (a) Domain structure of FD and substrates used in this study. Four conserved domains (C1 to C4) and the basic region/leucine-zipper (bZIP) are shown. Numbers indicate positions of amino acid residues. (b) In-gel kinase assay with full-length FD protein as a substrate. Extracts from whole aerial parts (Whole) or shoot apices (SA) of plants of indicated ages were analyzed. Kinase reactions were performed in the presence (+CaCl_2_) or absence (+EGTA) of Ca^2+^.

To identify the FD kinases responsible for phosphorylation of T282, we first performed an in-gel kinase assay using FD as a substrate and protein extracts from shoot apices and whole aerial parts of wild-type plants (Columbia-0 (Col)) as kinase sources. For the extracts, plant materials were harvested at three time points around the floral transition (Day 7, 10, and 14 after transfer of stratified seeds to the growth chamber) in our growth conditions. The in-gel kinase assay with the full-length FD protein as a substrate (Fig. 1a) produced two kinase signals. Of the two signals, a 55–60 kDa signal was calcium dependent while a 40–45 kDa signal was not (Fig. 1b). Under our long day (LD) condition, *AP1* expression was detected on Day 10 but not Day 7 (Supplementary Fig. S1a). TFL1 forms a complex with FD to prevent plants from flowering through the transcriptional repression of floral meristem identity genes including *AP1*^25^. Therefore, it is likely that phosphorylation of FD occurs before the onset of *AP1* expression. The results of the in-gel kinase assay with FD were consistent with this. To confirm the specificity of the phosphorylation, an *in vitro* kinase assay was performed with a C4 peptide containing T282 (amino acid residues 265–285; Fig. 1a) fused to glutathione *S*-transferase (GST). There are two potential phosphorylation sites, threonine residues at 276 (T276) and 282 (T282) in the C-terminal C4 domain of FD. Threonine-to-alanine substitutions of either T282 alone (T282A) or both T276 and T282 (T276A, T282A) abolished phosphorylation, while T276 alone (T276A) did not (Supplementary Fig. S1b). These results indicate that T282, but not T276, was phosphorylated by the protein kinase activity in the extract. To confirm the phosphorylation-dependent interaction with FT and 14-3-3, a non-phosphorylatable (T282A) and a phospho-mimic (threonine-to-glutamate substitution of T282; T282E) mutant of FD (mFD) were constructed. Wild-type (WT) FD and phospho-mimic T282E mFD interacted with FT in yeast and tobacco cells (Supplementary Fig. S1c, Fig. S1d). However, non-phosphorylatable T282A mFD failed to interact with FT, as previously reported^5^ (Supplementary Fig. S1c, Fig. S1d).

### Calcium-dependent protein kinase phosphorylates FD T282

**Figure 2:**
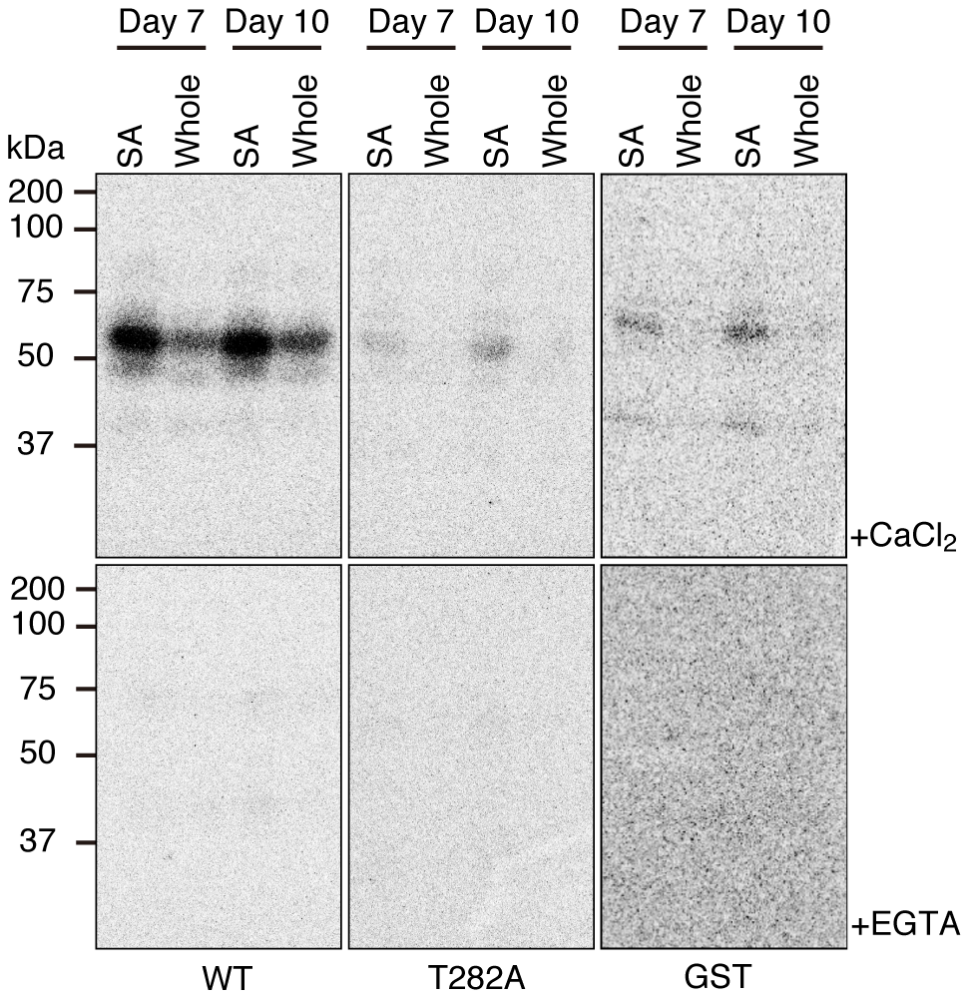
Kinase activity on FD T282 in protein extracts from shoot apices. In-gel kinase assay with wild-type (WT) or T282A versions of C4 peptide (Fig. 1a) fused to GST or GST alone as a substrate. Extracts from shoot apices (SA) or whole aerial parts (Whole) of plants of indicated ages were analyzed. Kinase reactions were performed in the presence (+CaCl_2_) or absence (+EGTA) of Ca^2+^.

For further biochemical characterization of kinase activity, we performed the in-gel kinase assay with WT and T282A versions of C4 peptide fused to GST (Fig. 1a). A calcium dependent signal of 55–60 kDa was detected with WT peptide, but not with T282A peptide or GST alone (Fig. 2). The presence of weak signals in the in-gel kinase assay without added substrates suggests that the similar weak signals in T282A and GST were derived from autophosphorylation of the kinases (Supplementary Fig. S2). Two types of calcium-regulated protein kinases are known in Arabidopsis. One is CDPK, and the other is calcineurin B-like proteins (CBL)-interacting protein kinase (CIPK). CIPK requires CBL, a calcium-binding protein, for its activity. The in-gel kinase assay can detect only protein kinases without a requirement of protein co-factors for its activity, but not those requiring protein co-factors. Therefore, our results suggest that the FD kinase(s) belong to CDPK.

**Figure 3:**
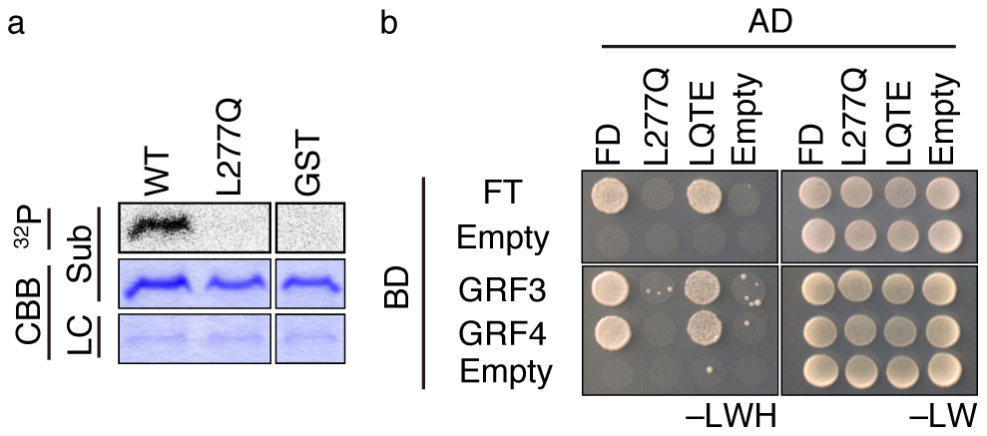
Effect of sequence alterations in C4 domain on phosphorylation and protein interaction. (a) *In vitro* kinase assay with wild-type (WT) or L277Q versions of C4 peptide (Fig. 1a) fused to GST or GST alone as a substrate.^32^P and CBB panels show autoradiography and Coomassie Brilliant Blue (CBB) staining images of parts of the gel. Substrate (Sub) and loading control (LC, showing a band around the molecular mass of RubisCO large subunit) panels indicate that similar amounts of substrates and protein extracts from shoot apices of Day 7 plants, respectively, were used in the experiment. (b) Effect of sequence alterations in the C4 domain on the interaction with FT and 14-3-3s, examined by yeast two-hybrid assay. Wild-type (FD), L277Q, and LQTE (L277Q T282E) versions of FD were tested for an interaction with FT and two isoforms of 14-3-3, GRF3 (14-3-3 psi) and GRF4 (14-3-3 phi). AD and BD indicate the activation domain and DNA binding domain, respectively, of GAL4. –LWH and –LW indicate selective (SCD –Leu, –Trp, –His) and non-selective (SCD –Leu, –Trp) medium, respectively.

To test whether CDPK phosphorylates FD T282, we analyzed substrate sequence specificity of protein kinases against FD C-terminal peptides. CDPK requires a hydrophobic amino acid residue at the −5 position for phosphorylation, especially leucine^32^. In other words, CDPK recognizes an L-X-R/K-X-X-S/T sequence and phosphorylates serine or threonine within this motif. The C-terminal sequence of L-Q-R-S-S-T in FD and its orthologous proteins conforms to this rule (Supplementary Fig. S3a). Thus, if CDPK is responsible for phosphorylation of FD T282, substitution of a leucine at the −5 position (L277) to glutamine (L277Q) should abolish phosphorylation. An *in vitro* kinase assay with a L277Q substitution in the C4 peptide (Fig. 1a) as a substrate showed that protein kinases in extracts of shoot apices failed to phosphorylate the C4 peptide with L277Q (Fig. 3a). Furthermore, L277Q mFD failed to interact with FT and 14-3-3s (GRF3 and GRF4) in yeast two-hybrid assays (Fig. 3b). As expected, phospho-mimic substitution (T282E) in L277Q mFD (termed LQTE) resulted in restoration of interaction with FT and 14-3-3 (Fig. 3b). These results indicate that L277 is crucial for phosphorylation of T282 and thereby the interaction with FT and 14-3-3s. To evaluate the activity *in planta*, we generated and analyzed transgenic plants expressing various mutant FD proteins under the control of the cauliflower mosaic virus (CaMV) *35S* RNA (*35S*) promoter in an *fd-1* background. In contrast to wild-type FD and phospho-mimic mutant FD (T282E mFD and LQTE mFD), which were able to rescue *fd-1*, non-phosphorylatable T282A mFD failed to rescue *fd-1*, and L277Q mFD showed reduced complementation ability (Supplementary Fig. S3b). Thus, the complementation tests indicate the importance of L277 for FD function *in planta*. Taken together, the biochemical characterization of substrate specificity (Fig. 3) and *in planta* complementation assays (Supplementary Fig. S3b) strongly suggest that CDPK phosphorylates FD T282 and is required for the FD-FT florigen protein complex formation.

### Subcelluar localization of FD and CDPKs

Subcellular localization of wild-type and mutant FD was examined using transient expression in *Nicotiana benthamiana*. A previous study showed that FD is localized in the nucleus and forms a complex with FT in the nucleus^5^. Wild-type FD, phospho-mimic T282E mFD, and non-phosphorylatable T282A mFD expressed as fusion proteins with enhanced yellow fluorescent protein (EYFP) were localized in the nucleus (Supplementary Fig. S4a). These results indicate that FD is constitutively localized in the nucleus in a phosphorylation-independent manner. Hence FD is likely to be phosphorylated in the nucleus by CDPKs present therein. To examine the subcellular distribution of CDPK proteins, expression plasmids encoding full-length CDPKs as EYFP-fusion proteins under the control of the *35S* promoter were introduced into *N. benthamiana* leaf epidermal cells by *Agrobacterium*-mediated transient transformation. Three different patterns of localization were observed. Fluorescence from EYFP-fused CPK3, 4, 5, 6, 11, 12, 26, 27, 31, and 33 was observed in the nucleus and cytoplasm (Supplementary Fig. S4b). Previously reported localization of CPK1 in peroxisomes and lipid bodies^33^,^34^ was confirmed (Supplementary Fig. S4b). The remaining CDPKs were localized in the plasma membrane (Supplementary Fig. S4b). It should be noted that while our assay was based on a transient expression system in tobacco cells, CPK localization patterns observed in our study were quite consistent with previous reports that exploited transient expression and/or stable expression systems in *Arabidopsis* cells^33^,^34^,^35^,^36^,^37^,^38^.

Ten CDPKs present in the nucleus were further analyzed together with a plasma-membrane localized CPK32 as a negative control.

### CPK4, CPK6, and CPK33 efficiently phosphorylate FD T282

**Figure 4:**
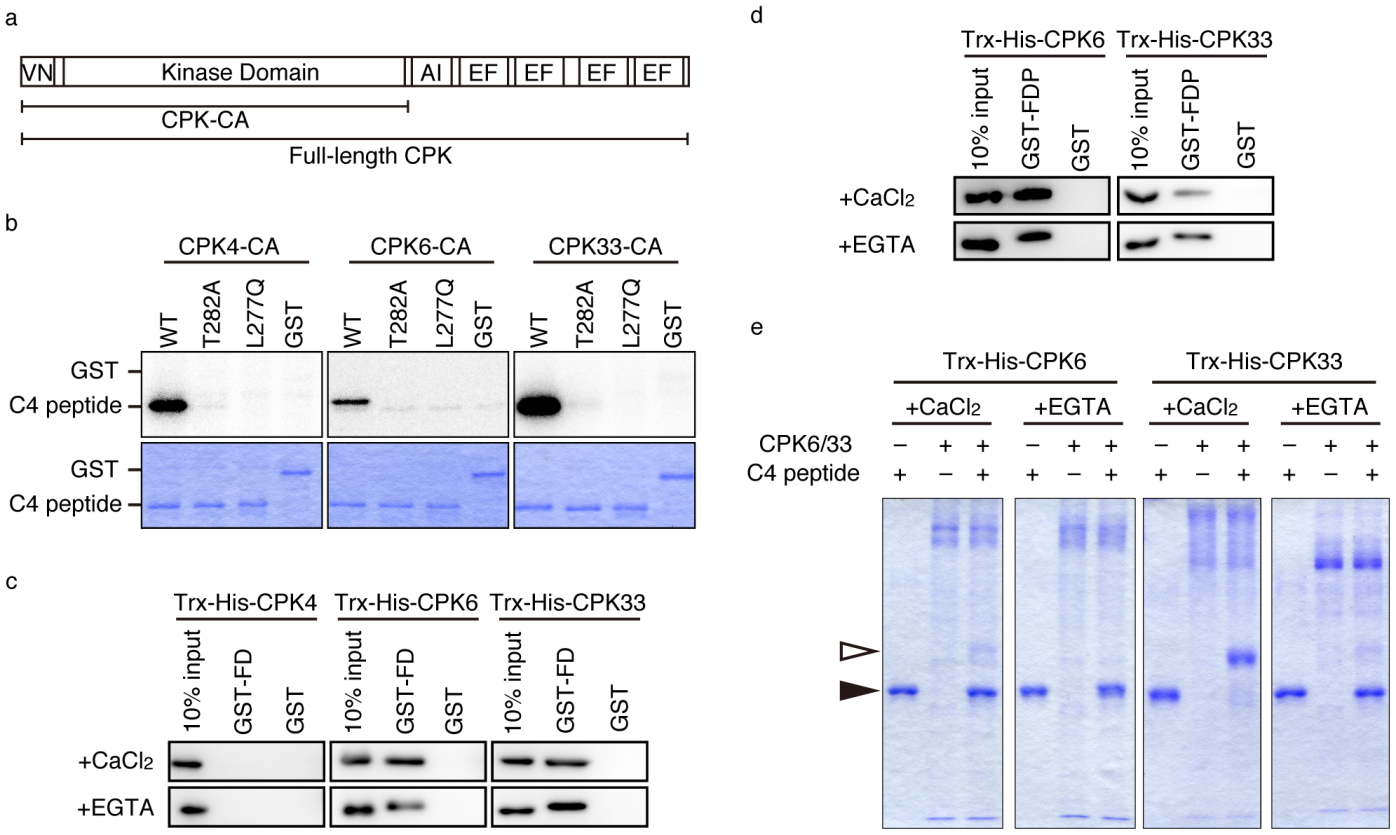
Biochemical characterization of candidate CDPKs. (a) Domain structure of CDPK and fragments used in this study. VN: variable N-terminal domain, AI: auto-inhibition domain, EF: EF hand motif. CPK-CAs were constructed based on published information^38^ and primers used for cloning are shown in Table S2. (b) *In vitro* phosphorylation of FD T282 by CPK-CAs. Wild-type (WT), T282A, and L277Q versions of C4 peptide (Fig. 1a) or GST were used as substrates. Lower panels (CBB staining) confirm similar amounts of substrates in the experiments. Uncropped images of autoradiograms are shown in Supplementary Fig. S5a. (c) *In vitro* pull-down assay. Trx-His-CPKs were pulled-down with either GST-FD or GST in the presence (+CaCl_2_) or absence (+EGTA) of Ca^2+^. Immunoblot with anti-His tag antibody. One-tenth volumes of the reactions were loaded in “10% input” lanes. Uncropped images are shown in Supplementary Fig. S6a (upper panels). Results of immunoblotting with anti-GST antibody are also shown in Supplementary Fig. S6a (lower panels). (d) *In vitro* pull-down assay. Trx-His-CPKs were pulled-down with either GST-FDP or GST in the presence (+CaCl_2_) or absence (+EGTA) of Ca^2+^. Immunoblot with anti-His tag antibody. Ten percent, by volume, of the reactions were loaded in “10% input” lanes. Uncropped images are shown in Supplementary Fig. S6b (upper panels). Results of immunoblotting with anti-GST antibody are also shown in Supplementary Fig. S6b (lower panels). (e) Calcium-dependency of kinase activity of CPK6 and CPK33. *In vitro* phosphorylation reactions used full-length purified CPK6 and CPK33. WT C4 peptide was used as a substrate. Phosphorylated and non-phosphorylated C4 peptides were separated by Phos-tag SDS-PAGE. White and black arrowheads indicate phosphorylated and non-phosphorylated C4 peptide, respectively.

*In vitro* kinase assays were performed to evaluate the enzymatic activity of candidate CDPKs on C4 peptide and truncated FD (tFD; amino acid residues 196–285) (Fig. 1a) as substrates (Supplementary Fig. S5). Constitutively active forms of CDPKs (CPK-CAs) lacking both the C-terminal auto-inhibition region and EF-hand motifs^38^ (Fig. 4a) were used for the assay. All the CPK-CAs except for CPK31 were expressed as soluble recombinant proteins and had kinase activity against a general substrate, myelin basic protein (MBP) (Supplementary Fig. S5a). Among the tested CDPKs, CPK4, CPK6, and CPK33 efficiently phosphorylated T282 in C4 peptide (Fig. 4b, Supplementary Fig. S5a). These three CDPKs and 5 other CDPKs that phosphorylated T282 in C4 peptide were further analyzed for their kinase activity against the tFD fragment containing the bZIP region for dimerization. CPK4, CPK6, and CPK33 efficiently phosphorylated T282 in tFD, as well. By contrast, the activity of CPK3, 5, 11, 27, and 32 was decreased or lost compared to the activity against C4 peptide (Supplementary Fig. S5b). These results suggest that CPK4, CPK6, and CPK33 are good candidates as FD kinases.

### CPK6 and CPK33 directly interact with FD

To investigate protein-protein interactions between FD and candidate CDPKs, *in vitro* pull-down assays were performed. Trx-His-CPK4, Trx-His-CPK6, and Trx-His-CPK33 were tested with either GST-FD or GST. CPK6 and CPK33 were co-precipitated with GST-FD in the presence of Ca^2+^ but not GST (Fig. 4c, Supplementary Fig. S6a; +CaCl_2_). These results indicate that CPK6 and CPK33 are able to interact directly with FD. Since co-precipitation with GST-FD was observed in the absence of Ca^2+^ (Fig. 4c, Supplementary Fig. S6a; +EGTA), the interaction does not require Ca^2+^. Similar calcium-independent, direct interaction was observed for a closely related protein, FD PARALOG (FDP)^5^,^6^,^25^,^39^ (Fig. 4d, Supplementary Fig. S6b).

### CPK6 and CPK33 depend on Ca^2+^ for the kinase activity

While CPK6 and CPK33 interacted with FD in a calcium-independent manner (Fig. 4c), the T282-directed kinase activity in extracts of shoot apices was clearly calcium dependent (Fig. 1b, Fig. 2). To test the calcium dependency of CPK6 and CPK33 for their kinase activity, we performed *in vitro* phosphorylation assays with the respective full-length CPKs against C4 peptide as a substrate. The assay was based on a protein mobility shift by Phos-tag SDS-PAGE, which can separate phosphorylated proteins based on the differential binding affinity to the attached phosphate groups^40^. Phosphorylated C4 peptides were detected only in the presence of Ca^2+^ (+CaCl_2_ condition) for both CPK6 and CPK33 (Fig. 4e). These observations of calcium-dependent kinase activity of CPK6 and CPK33 are consistent with the notion that these two kinases represent the phosphorylation activity in the shoot apex detected by the in-gel kinase assay (Fig. 1b, Fig. 2).

### CPK6 and CPK33 are present in the nucleus in shoot apical cells

**Figure 5:**
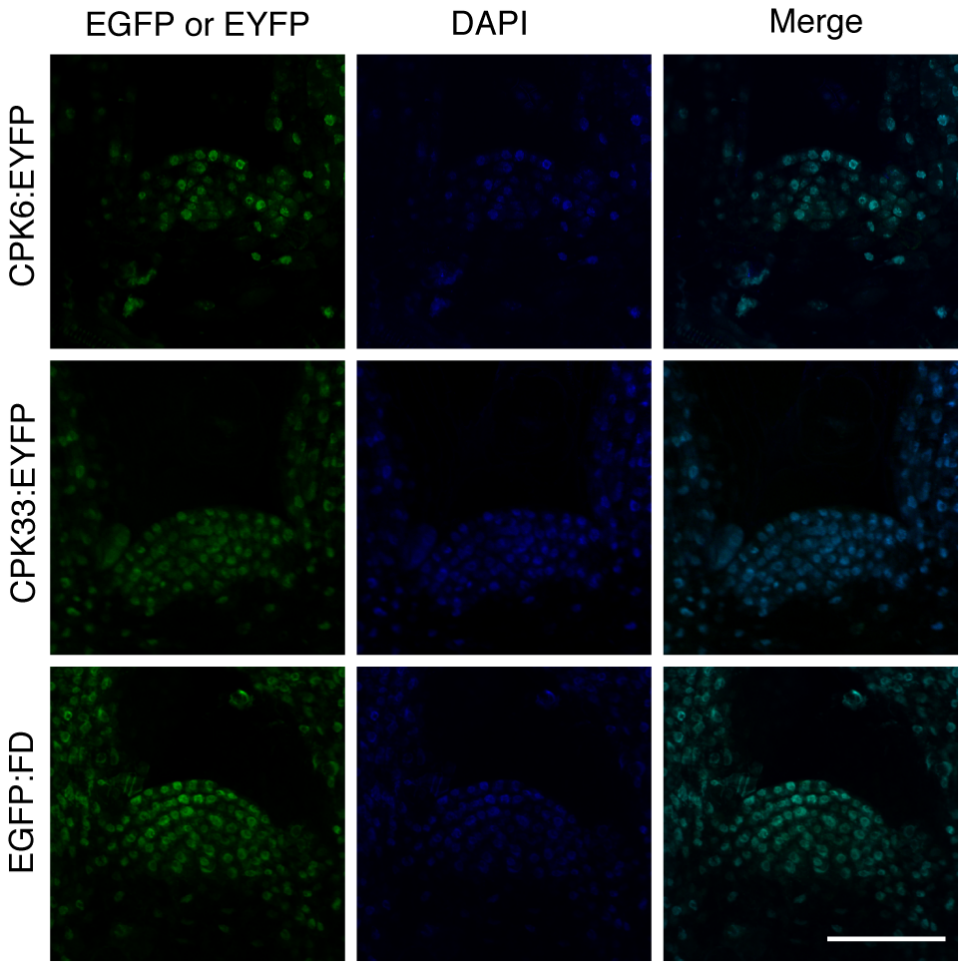
Spatial expression patterns and subcellular localization of FD, CPK6, and CPK33 in shoot apical meristem. Shoot apical meristem in 7-day-old plants of *pCPK6::CPK6:EYFP:3HA:His*, *pCPK33::CPK33:EYFP:3HA:His*, and *pFD::EGFP:FD; fd-1* are shown. EYFP (CPK:EYFP) or EGFP (EGFP:FD) fluorescence, DAPI fluorescence, and merged images are shown in the same magnification. Scale bar: 50 μm.

RT-PCR analysis and an Arabidopsis eFP browser search showed that both CPK6 and CPK33 are expressed in the shoot apex (Supplementary Fig. S7). To visualize the expression pattern and subcellular distribution, transgenic *Arabidopsis* plants carrying the respective CPKs fused to EYFP under the control of their own promoters (*pCPK6::CPK6:EYFP:3HA:His* and *pCPK33::CPK33:EYFP:3HA:His*) were generated. In both transgenic plants, fusion proteins were expressed in the shoot apical meristem, and EYFP fluorescence was detected in the nucleus (Fig. 5). As previously noted, FD was expressed in the shoot apical meristem and was localized in the nucleus, when examined with a similar construct to express FD fused to enhanced green fluorescent protein (EGFP) (*pFD::EGFP:FD; fd-1*) (Fig. 5). These results suggest that FD and two candidate CDPKs are all present in the nucleus of shoot apical cells. To observe co-localization of CPK33 and FD *in planta*, CPK33:EYFP and FD:mCherry fusion proteins were transiently co-expressed in leaf epidermal cells of *N. benthamiana*. Analysis of fluorescence intensity profiles showed that EYFP and mCherry signals overlapped in the nucleus (Supplementary Fig. S8).

**Figure 6:**
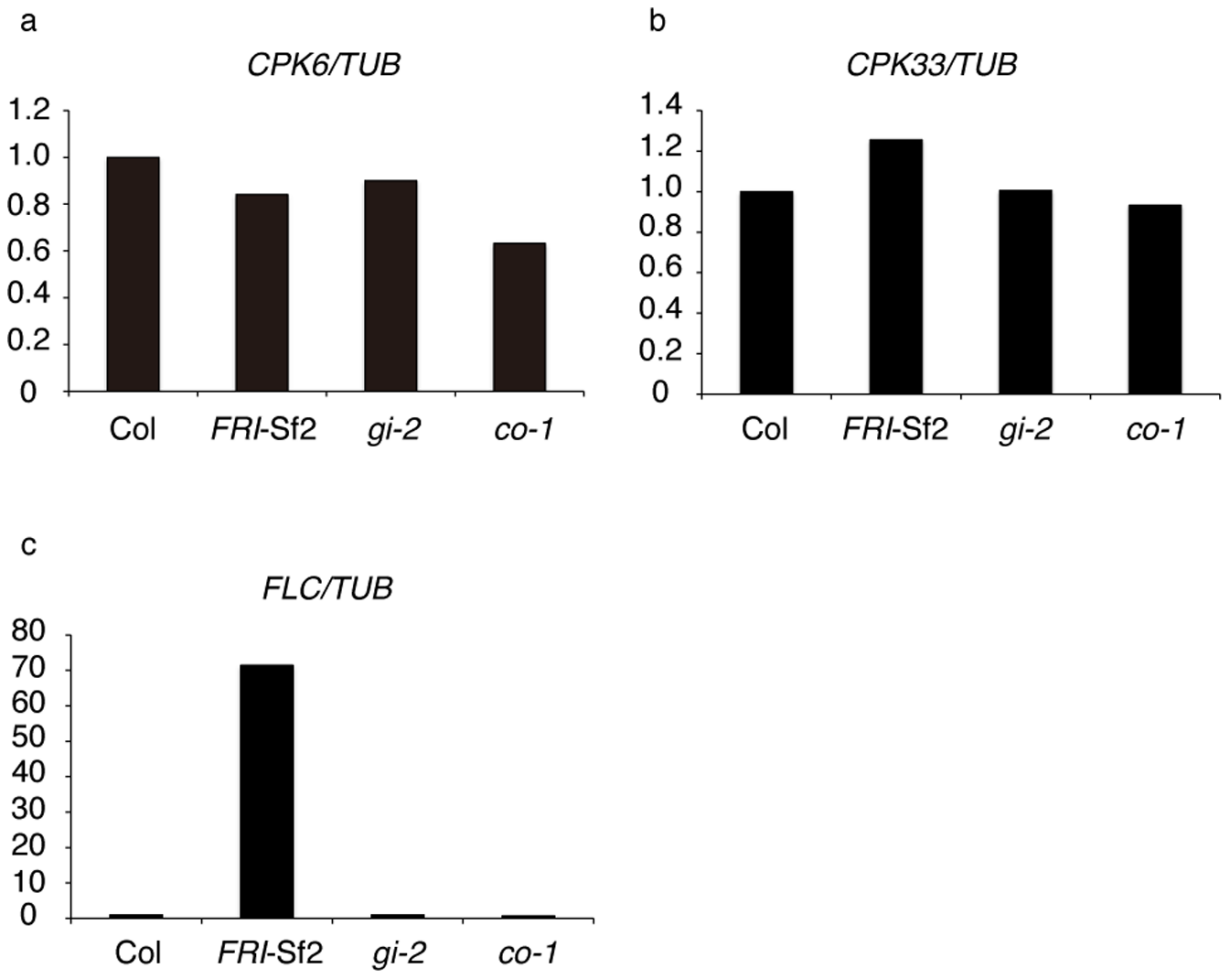
Expression of *CPK6* and *CPK33* in backgrounds with altered flowering regulators. Relative levels of *CPK6* (a), *CPK33* (b), and *FLC* (c) mRNA in shoot apices. Expression levels were quantified by quantitative RT-PCR analysis. *TUBULIN2/3* (*TUB*) was used as an internal control. Relative levels to that of Col (defined as 1) are indicated. Results of a single set of experiments are shown. Plants were grown under LD condition for 7 days. *FRI*-Sf2 is a Col line with a *FRI* allele (*FRI*-Sf2) from San Feliu-2 accession.

Expression of *CPK6* and *CPK33* in wild-type, *FRI*-Sf2 (San Feliu-2 allele of *FRIGIDA*), *gigantea* (*gi*), and *constans* (*co*) backgrounds were examined by quantitative reverse transcription-PCR (qRT-PCR). In a *FRI*-Sf2 background, expression of *FLOWERING LOCUS C* (*FLC*), a negative regulator of florigen production and response through repression of *FT* and *FD* expression^41^, was greatly up-regulated (Fig. 6). *GI* and *CO* are key components of the photoperiod pathway upstream of *FT* and *TSF*. Expression of *CPK6* and *CPK33* was affected in none of the tested backgrounds (Fig. 6), indicating that these genes are not under the control of these regulators of florigen production and response.

### *cpk33-1* displayed a late-flowering phenotype and enhanced *lfy-1* pehnotype

If CPK6 and CPK33 are responsible for FD phosphorylation *in planta*, their loss of function mutants are expected to show a late-flowering phenotype due to reduced florigen complex formation. However, since FD also forms a complex with other PEBPs acting as a flowering repressor, such as TFL1^23^,^25^, the expected late-flowering phenotype of these *cpk* mutants is likely to be attenuated through counteracting effects between promoters (FT and TSF) and repressors (TFL1, ATC, and BFT). Functional redundancy among CDPK species may also attenuate the phenotype. With these in mind, we examined the phenotype of T-DNA insertion mutants of *cpk6* (*cpk6-1*) and *cpk33* (*cpk33-1* and *cpk33-2*). *cpk4* (*cpk4-1*) was also included in the analysis (Supplementary Fig. S9). Transcripts spanning the entire full-length coding sequence of the corresponding genes were detected in none of the 4 *cpk* mutants (Supplementary Fig. S9c). Double mutants (*cpk6-1* *cpk33-1, cpk4-1* *cpk6-1*, and *cpk4-1* *cpk33-1*) were also analyzed for their flowering time. However, none of the single and double *cpk* mutants showed a late-flowering phenotype under LD condition (Supplementary Fig. S10a).

**Figure 7:**
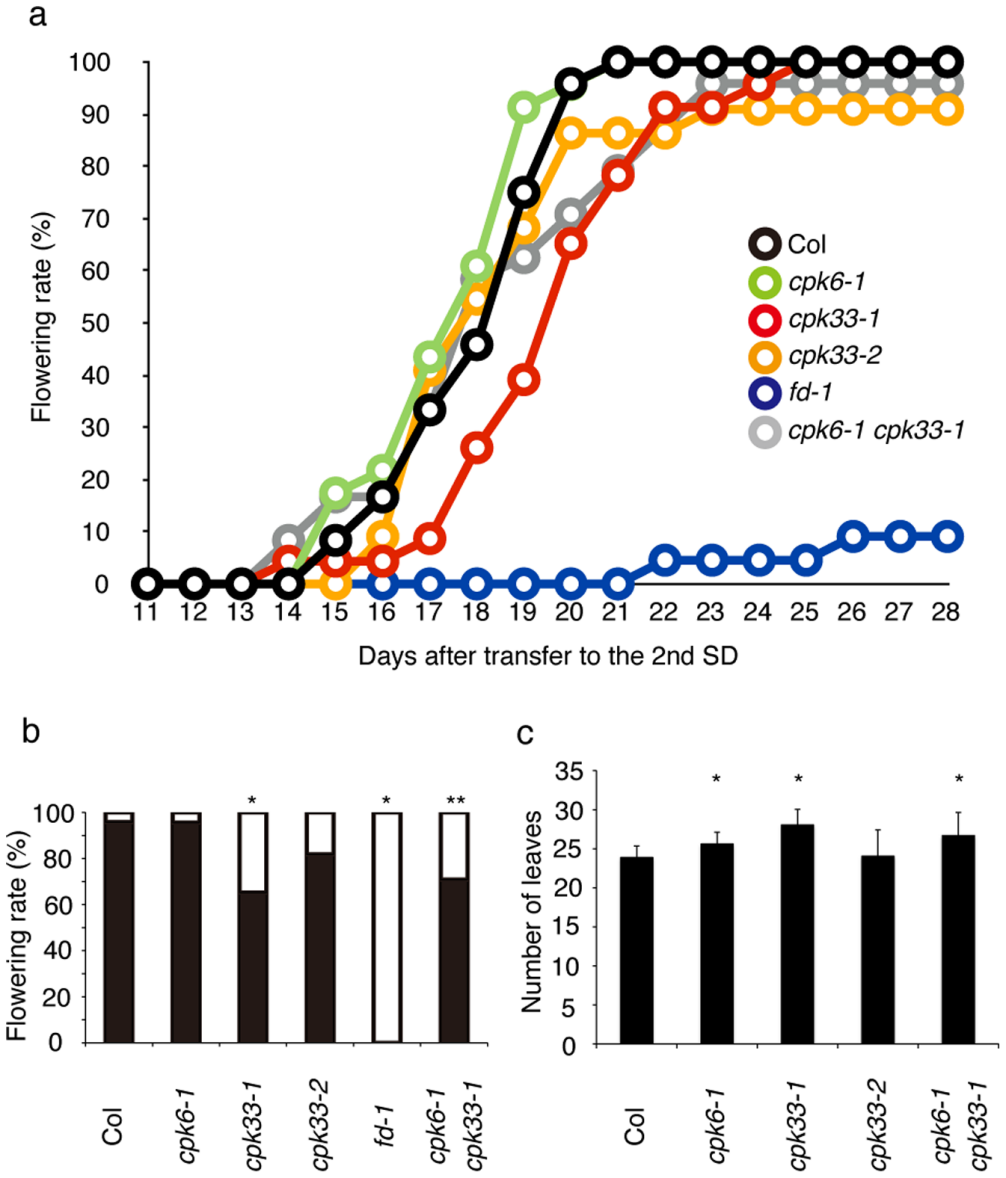
Effect of *cpk* mutations on flowering. Plants (*n* = 22–24) grown in SD for 3 weeks were treated by 4 LDs, transferred back to SD (Supplementary Fig. S11a), and were examined daily for flowering. The number of rosette and cauline leaves was counted after flowering. (a) Cumulative rate of flowering plants. The horizontal axis indicates days after transfer to the 2nd SD. (b) Percentage of flowering (black) and non-flowering (white) plants 20 days after transfer to the 2nd SD. Asterisks above bar indicate statistically significant difference from Col (*: *P* < 0.01, **: 0.01 < *P* < 0.05, Fisher's exact test). (c) Flowering time as measured by the number of leaves at flowering (mean ± SD). An asterisk above bar indicates statistically significant difference from Col (*P* < 0.001, Student's *t*-test [two-tailed test]). Note that there is not statistically significant difference between *cpk 33-1* and *cpk6-1 cpk33-1* (*P* > 0.07, Student's *t*-test).

To improve the sensitivity of detection of a subtle phenotypic effect by *cpk* mutations, we adopted the following growth conditions in which the amount of florigen signal, including FT induced by the photoperiod pathway, is reduced^7^,^42^ (Fig. S11). Plants were grown under a non-inductive short-day (SD) condition for 3 weeks and transferred to an inductive LD condition and kept for 4 days. After 4 days of LD growth, plants were transferred back to the SD condition (Fig. S11a) and the cumulative rate of flowering plants was scored daily. The increase in the cumulative flowering rate was significantly delayed in *cpk33-1*, while it was not affected in *cpk6-1* (Fig. 7a, Fig. 7b). Measurement of the number of leaves confirmed that *cpk33-1* caused delayed floral transition (Fig. 7c). *cpk6-1* and the *cpk6-1 cpk33-1* double mutation also resulted in a delayed flowering phenotype (Fig. 7b, Fig. 7c). These results suggest that CPK33 is involved in the regulation of floral transition. CPK6 may have a minor contribution which is largely masked in the presence of CPK33 activity. As anticipated from the weak phenotype and functional redundancy, neither single nor double mutants showed a significant decrease in kinase activity for FD T282 in protein extracts from shoot apices (Supplementary Fig. S10b). The observation that neither *cpk6-1* nor *cpk33-1* enhanced the late-flowering phenotype of *fd-1* (Supplementary Fig. S10a) is consistent with the notion that the two CDPKs act in the same pathway as FD.

**Figure 8:**
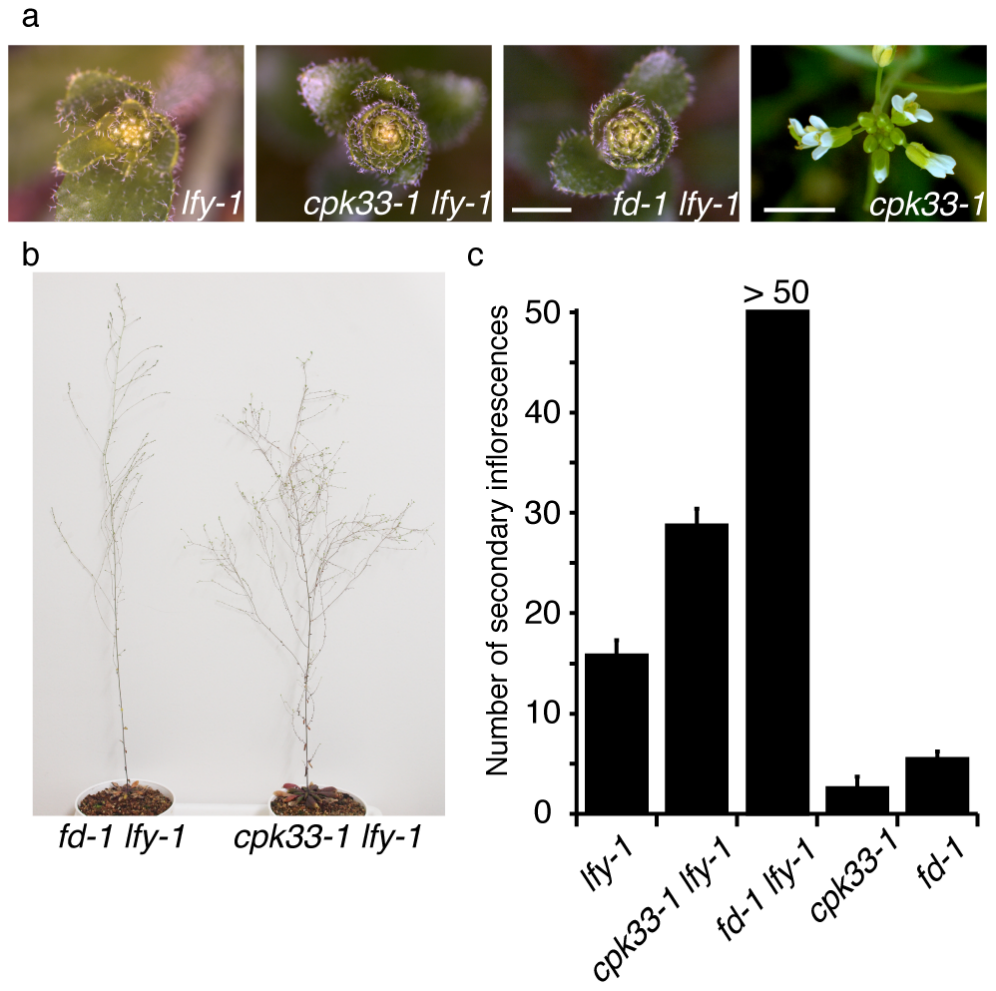
Enhancement of *lfy* phenotype by *fd* and *cpk33*. (a) Top view of the primary inflorescence at the stage of 5-cm height. Scale bar: 2 mm. Left three panels share the same scale bar. (b) Shoot architecture of a representative plant of 2-month-old *fd-1* *lfy-1* and *cpk33-1lfy-1*. (c) The number of secondary inflorescences formed before making the first solitary flower or flower-like structure. Number of plants for each genotype are as follows. *lfy-1*; *n* = 7, *cpk33-1 lfy-1*; *n* = 5, *fd-1 lfy-1*; *n* = 9, *cpk33-1*; *n* = 8, *fd-1*; *n* = 8. Note that the number of secondary inflorescences of Col and *cpk33-1* is indistinguishable (Col; 2.79 ± 0.66, *cpk33-1*; 2.63 ± 0.65, *n* = 25 for both genotypes. *P* > 0.3, Student's *t*-test).

It was previously shown that *fd* and *ft* enhanced flower-to-shoot conversion phenotype of *lfy* through reduction of *AP1* expression^5^,^6^. If CPK33 is involved in complex formation between FD and FT, a similar enhancement of *lfy* phenotype by *cpk33* mutation is expected. In *cpk33-1 lfy-1* mutant, the number of secondary inflorescences (leafy shoots) formed before making solitary flowers was greatly increased as compared to *lfy-1*, indicating the enhancement of flower-to-shoot conversion phenotype (Fig. 8). As expected from functional redundancy, observed enhancement was weaker than that of *fd-1* (Fig. 8).

## Discussion

The C-terminal C4 domain of FD and its orthologous proteins is conserved in a wide variety of flowering plant species^16^,^17^,^18^,^19^,^20^,^21^ (Supplementary Fig. S3a), and a threonine or serine residue therein to be phosphorylated is crucial for complex formation with PEBPs involved in the regulation of flowering^5^,^6^,^19^,^27^,^30^. Therefore, demonstration of phosphorylation and identification of responsible protein kinase(s) are important for understanding the florigen action at the shoot apical meristem^30^.

Biochemical characterization of a protein kinase activity present in the extract from shoot apices revealed the following characteristics. The activity is detectable by an in-gel kinase assay (Fig. 1b, Fig. 2). It requires Ca^2+^ for phosphorylation of FD T282 (Fig. 1b, Fig. 2), and its substrate preference conforms to the CDPK consensus sequence, L-X-R/K-X-X-S/T (Fig. 3, Supplementary Fig. S3). These properties suggest that the FD kinase(s) belong to the CDPK family. Candidate CDPKs were selected through a two-step screening of subcellular localization and activity assays. Of the candidate CDPKs, CPK6 and CPK33 were expressed in the shoot apical meristem and were present in the nucleus (Fig. 5, Supplementary Fig. S7), directly bound FD (Fig. 4c, Supplementary Fig. S6a), and efficiently phosphorylated FD (Fig. 4b, Supplementary Fig. S5). Furthermore, a loss of function *cpk33* mutant showed a late-flowering phenotype (Fig. 7) and enhanced *lfy-1* phenotype (Fig. 8). Based on these observations, we concluded that CDPKs, including CPK6 and CPK33, are responsible for the phosphorylation of FD T282 and complex formation via 14-3-3 with FT in the shoot apex to regulate flowering. Clades of CDPKs including those to which these two belong are conserved among flowering plants^43^,^44^. Thus, CDPK is likely to constitute a part of the conserved regulatory module involving the FD-FT protein complex in flowering plants.

Of the two loss-of-function mutants of CPK33, *cpk33-1* had a T-DNA insertion in the 2nd exon, which encodes a kinase domain (Supplementary Fig. S9), and is expected to be a null allele (Supplementary Fig. S9c). A weak late-flowering phenotype of *cpk33-1* implies the presence of other CDPKs, such as CPK6, redundantly involved in FD phosphorylation, although CPK33 may be the major protein kinase based on its strong kinase activity with FD *in vitro*. To assess the relative contribution among candidate CDPKs to the phosphorylation of FD T282, the in-gel kinase assay was performed with protein extracts from shoot apices of *cpk* mutants. However, a clear impairment of kinase activity on FD T282 was observed in none of the tested *cpk* single and double mutants (Supplementary Fig. S10b). It is likely that the activity of other CDPKs present in the extract, which may not necessarily be involved in the FD phosphorylation *in planta*, compensated for the loss of tested CDPKs in the assay. Alternatively, yet unidentified CDPKs might be redundantly involved in the phosphorylation. In our subcellular localization analysis, closely related pairs of CDPKs showed a similar localization pattern (e.g. CPK4 and CPK11 were both present in the nucleus and cytosol; Supplementary Fig. S4b) and these pairs function redundantly in many cases (e.g. CPK17 and CPK34, CPK4 and CPK11^45^,^46^). By contrast, however, CPK33 and its closest paralogous protein, CPK9 (Supplementary Fig. S10c) showed different subcellular distribution patterns. CPK9 was exclusively localized in the plasma membrane (Supplementary Fig. S4b), as previously described^33^,^47^. Therefore, it is unlikely that CPK9 represents a yet unidentified redundant FD kinase.

As mentioned above, FD is involved in both promotion and repression of flowering via phosphorylation-dependent complex formation with either the FT subfamily (florigen; FT and TSF) or TFL1 subfamily (anti-florigen; TFL1, ATC, and BFT) of PEBPs^5^,^6^,^24^,^25^,^26^,^27^,^28^. In addition, there exits a closely related gene, *FDP*. Although controversial reports on the role of FDP in flowering have been published^25^,^39^, FDP shares a similar C-terminal sequence with FD (Supplementary Fig. S3a) and interacts with FT and TFL1^5^,^6^,^25^. Since FDP can directly interact with CPK33 (Fig. 4d, Supplementary Fig. S6b), it is likely to be phosphorylated by CPK33 and form a complex with FT and TFL1 in a phosphorylation-dependent manner. Therefore, a weak late-flowering phenotype of *cpk33-1* may also be in part due to a combined effect on florigen and anti-florigen complex formation of FD and FDP.

FD belongs to the A-group bZIP transcription factors which includes those involved in abscisic acid signaling^48^. Studies of these bZIP transcription factors demonstrated that their multiple phosphorylation by protein kinases of the SnRK2 family is important for transactivation of their target genes^49^,^50^. Interestingly, the in-gel kinase assay demonstrated that FD is also phosphorylated by calcium-independent protein kinase(s), the highest activity of which was present in the extracts of shoot apices from a later stage (Day 10) than the peak of calcium-dependent protein kinase(s) specific for T282 (Fig. 1b). Phosphorylation by this calcium-independent protein kinase activity might be important for recruiting proteins such as co-activators and formation of a transcriptional activation complex. Further investigation such as phosphorylation site mapping and mutational analysis of FD will be important for testing this hypothesis.

Identification of calcium-dependent protein kinases as an important component in regulation of flowering, responsible for FD phosphorylation and florigen complex formation, prompts us to re-evaluate the role of calcium signaling in the regulation of flowering proposed by observations of calcium accumulation in the shoot apex in *Sinapis alba*^51^. The investigation of calcium dynamics during the floral transition by modern imaging techniques such as a calcium biosensor, Yellow Cameleon, will certainly contribute to the understanding of the molecular nature underlying regulation of flowering at the shoot apex.

## Methods

### Plant materials and growth conditions

Columbia-0 (Col) was used as the wild type. *fd-1* (Q86 to Stop) in the Col background and *pFD::EGFP:FD; fd-1* were previously described^5^. *FRI-*Sf2, *gi-2*, *co-1* and *lfy-1* are in the Col background. Note that the original *co-1* in the Landsberg *ER*^+^ background was backcrossed 6 times with Col to obtain the *co-1* line used in this study. T-DNA insertion lines of CPK4 (*cpk4-1*; SALK_081860), CPK6 (*cpk6-1*; SALK_093308), and CPK33 (*cpk33-1*; SALK_059467, *cpk33-2*; SALK_036145) were obtained from the Arabidopsis Biological Resource Center (ABRC) and backcrossed once with Col. *pCPK6::CPK6:EYFP:3HA:His* and *pCPK33::CPK33:EYFP:3HA:His* were generated in this study. Half-strength Murashige and Skoog (MS) medium with 0.5% sucrose containing 0.8% agar was used for aseptic culture. Seeds were stratified at 4°C for 2–3 days and then transferred to 22°C (defined as Day 0). Plants were grown under LD (16 h light/8 h dark) conditions with white fluorescent lights (~60 μmol m^−2^s^−1^) or SD (8 h light/16 h dark) conditions with white fluorescent lights (~100 μmol m^−2^s^−1^).

### Protein expression and purification

*Escherichia coli* strain BL21 (DE3) or Rosette2 (DE3) were transformed with plasmids and cultured at 37°C. When the absorbance at 600 nm of the cultures reached 0.4, IPTG and ethanol were added to a final concentration of 1 mM and 2%, respectively for induction, and cells were cultured at 15°C for 16–24 h. Cells were harvested and disrupted by sonication to obtain cell free extracts. Recombinant full-length FD protein was purified from the inclusion body under denaturation conditions using Ni-NTA agarose (QIAGEN) according to the manufacture's instructions. Recombinant GST-tagged and His-tagged proteins were purified from cell-free extracts with a GSTrap FF column (GE Healthcare) and HisTrap HP column (GE Healthcare), respectively. The purities of the recombinant proteins were verified by SDS-PAGE and staining with Rapid Stain CBB (Nacalai tesque).

### In-gel kinase assay

The in-gel kinase assay was performed as described^52^ with the full-length FD and GST-fused C4 peptides as substrates in a 10% SDS-polyacrylamide gel (2.5 mg/gel). Plant tissues were ground in liquid nitrogen and homogenized in TG150 buffer (25 mM Tris-HCl pH 7.5, 10 mM EGTA, 10 mM EDTA, 150 mM NaCl, 10% Glycerol, 0.1% Triton X-100, 1 mM DTT, 20 mM β-glycerophosphate, 1 mM Na_3_VO_4_, 1 mM NaF, and Complete EDTA-free protease inhibitor (Roche)). The homogenates were centrifuged at 15,000 rpm for 20 min at 4°C. The resultant supernatant was used as a protein extract. Thirty μg of protein extracts from Day 7, Day 10, and Day 14 Col plants were denatured and subjected to electrophoresis. SDS was removed by successive washing with SDS-removal buffer I (50 mM Tris-HCl, pH 8.0, 20% isopropanol) and II (50 mM Tris-HCl, pH 8.0, 5 mM 2-mercaptoethanol) for 30 min twice, respectively. After denaturation of the gel with denaturation buffer (50 mM Tris-HCl, pH 8.0, 5 mM 2-mercaptoethanol, 6 M guanidine hydrochloride) for 1 h, the gel was washed twice with renaturation buffer (SDS-removal buffer II containing 0.04% Tween 40) for 30 min, then was incubated in the same buffer at 4°C for 16 h, and then a final wash for 30 min. Subsequently, the gel was incubated with kinase reaction buffer (40 mM HEPES-KOH, pH 7.5, 15 mM MgCl_2_, 2 mM DTT) for 30 min at room temperature. Then, 2.5 μl of [γ-^32^P] ATP (6,000 Ci/mmol) and 5 μl of 100 mM ATP were added to the reaction buffer and incubated for 1 h at room temperature. For the calcium-dependence assay, either CaCl_2_ (0.5 mM) alone or both CaCl_2_ (0.1 mM) and EGTA (0.5 mM) were added to the kinase reaction buffer, respectively. After the 1 h incubation, the gel was washed extensively with stop solution (5% trichloroacetic acid, 1% pyrophosphate) and dried. Autoradiography was performed with Imaging Plate and FLA-4000 (Fuji Film).

### *In vitro* kinase assay

The *in vitro* kinase assay was performed as previously described^53^. For the kinase assay, 2 μg of purified substrate proteins were mixed with 4 μl of 5× kinase buffer (40 mM HEPES-KOH, pH 7.5, 15 mM MgCl_2_, 0.5 mM CaCl_2_, 2 mM DTT), 5 μCi [γ-^32^P] ATP, 50 μM ATP and 5 μg of extracts from shoot apices as a source of protein kinases or 2 μg of purified CDPKs. Reactions were started by adding protein kinases, and were terminated by adding 5 μl of 5× SDS-sample buffer. Samples were boiled at 96°C for 5 min, spun briefly, and subjected to SDS-PAGE. Autoradiography was performed with Imaging Plate and FLA-4000 (Fuji Film). For the calcium-dependence assay, either CaCl_2_ (0.5 mM) alone or both CaCl_2_ (0.1 mM) and EGTA (0.5 mM) were added to the kinase reaction buffer. Reactions were done without addition of [γ-^32^P] ATP and were subjected to electrophoresis on 10% polyacrylamide gel containing 20 μM Phos-tag acrylamide (Wako Pure Chemicals), followed by staining with rapid stain CBB.

### *In vitro* pull-down assay

Bacterial lysates containing GST-tagged proteins were incubated with 20 μl of glutathione sepharose 4B resin (GE Healthcare) for 30 min at 4°C. Resin was washed 5 times with phosphate buffered saline (PBS) and once with pull-down buffer plus (25 mM HEPES-KOH pH 7.5, 100 mM NaCl, 1 mM CaCl_2_, 1 mM DTT, 0.1% NP-40) or pull-down buffer (25 mM HEPES-KOH pH 7.5, 100 mM NaCl, 1 mM EGTA, 1 mM DTT, 0.1% NP-40). Five μg of purified Trx-His-CPK proteins and 400 μl of pull-down buffer plus (or pull-down buffer) were added to each resin and the mixture was gently stirred for 2 h at 4°C. The resins were washed 3 times with the same buffer and proteins were eluted with SDS-sample buffer. Eluted proteins were subjected to SDS-PAGE and immunoblot analyses. Trx-His-CPK proteins were detected with Anti-His antibody (CST) and an appropriate HRP-labeled secondary antibody using ECL prime (GE Healthcare).

Detailed procedures of plasmid construction, *Arabidopsis* and tobacco transformation, yeast two-hybrid assay, RT-PCR and qRT-PCR analysis, analysis of spatial expression pattern are described in Supplementary Information.

## Author Contributions

N.K., M.E., Y.M. and T.A. designed the experiments. N.K. and M.S. performed experiments. N.K., M.E. and T.A. analyzed the results. N.K. and T.A. wrote the paper.

## Supplementary Material

Supplementary Information Supplementary Information
