# Supplementary material for: Calcium-dependent protein kinases responsible for the phosphorylation of a bZIP transcription factor FD crucial for the florigen complex formation
Source: Sci Rep. 2015 Feb 9;5:8341. doi: 10.1038/srep08341 (PMC4321167; doi:10.1038/srep08341)
Supplement: Supplementary Information [file srep08341-s1.pdf]

Supplementary Information

Title: Calcium-dependent protein kinases responsible for the phosphorylation of a bZIP transcription factor FD crucial for the florigen complex formation

Author: Nozomi Kawamoto[1], Michiko Sasabe[2,3], Motomu Endo[1], Yasunori Machida[2], and Takashi Araki[1]

[1]Division of Integrated Life Science, Graduate School of Biostudies, Kyoto University, Yoshida-Konoe-cho, Sakyo-ku, Kyoto, 606-8501, Japan

[2]Division of Biological Science, Graduate School of Science, Nagoya University, Furo-cho, Chikusa-ku, Nagoya, 464-8602, Japan

[3]Present address: Department of Biology, Faculty of Agriculture and Life Science, Hirosaki University, Hirosaki 036-8561, Japan

Contact address

Corresponding author: Takashi Araki (taraqui@lif.kyoto-u.ac.jp)

Materials and Methods

Plasmid construction

All the plasmid constructs were generated using standard molecular biology methods. Lists of plasmids and primers used in the construction are presented in Table S1 and Table S2.

FD-His: FD was cloned into *Nde* I/*Sal* I sites of pET41a.

GST-oligo FDs: Single-strand oligo DNA fragments (corresponding to M265-F285 of FD) were hybridized and ligated into *Spe* I/*Xho* I sites of pET41a.

GST-tFDs: Wild-type and T282A versions of tFD (V196-F285) were cloned into *Spe* I/*Sal* I sites of pET41a.

GST-FD: FD ORF was cloned into *Bam*H I/*Sal* I sites of pGEX6P-1.

GST-FDP: FDP ORF was cloned into *Bam*H I/*Sal* I sites of pGEX6P-1.

FD:mCherry: mCherry ORF was cloned into the *Sac* II site of pPZP211/35S to generate pPZP211/35S::mCherry. Then, FD ORF was cloned into *Bam*H I and *Sma* I sites of pPZP211/35S::mCherry to generate the FD:mCherry translational fusion construct.

CPK-CA: ORF of each CPK gene without the C-terminal auto-inhibition region and

EF-hand motifs was amplified by PCR and cloned into pET32a by the In-Fusion cloning system (Clontech). For CPK4, CPK6, and CPK33, full-length ORF was also cloned by the In-Fusion cloning system.

CDPK library (full length): Each CPK gene was amplified by PCR and cloned into pPZP211/35S::EYFP:3HA:His vector by the In-Fusion cloning system. This vector was generated by the following procedure. 3HA:His-coding single-strand DNA fragments were hybridized and ligated into the *Sac* II site of pPZP211/35S to generate pPZP211/35S::3HA:His. EYFP was cloned by the In-Fusion cloning system into *Sac* I/*Sac* II sites of pPZP211/35S::3HA:His.

Yeast two-hybrid constructs: Wild-type and mutant versions of FD, FT, and 14-3-3s were cloned into pCUY[54] and pGAD424, respectively into the following restriction enzyme sites: FD (*Bam*H I/*Sal* I); mutant FDs (*Sma* I/Sal I); FT (*Sma* I/*Sal* I); GRF3 (14-3-3 Psi) (*Bam*H I/*Pst* I); and GRF4 (14-3-3 Phi) (*Sma* I/*Bam*H I).

BiFC constructs: YN-T282E was ligated into *Xba* I/*Sac* I sites of pBI121.

Plant transformation constructs: the *35S* promoter of pPZP211/35S::CPK6:EYFP:3HA:His and pPZP211/35S::CPK33:EYFP:3HA:His was

replaced by the promoters of *CPK6* and *CPK33*, respectively, by the In-Fusion cloning system with *Hin*d III and *Xba* I sites. The genomic fragments 3,001-bp and 3,599-bp upstream of the start ATG codon were used as promoter regions of *CPK6* and *CPK33*, respectively.

fd-1 complementation test: Wild-type and mutant versions of FD were cloned into *Bam*H I/*Sac* II sites of pENTR1A and LR reaction to pFAST-G02.

Arabidopsis transformation

The constructs in binary vectors were introduced into *Agrobacterium tumefaciens* strain GV3101::pMP90 and transformed into *Arabidopsis* by the floral dip procedure. For the *fd-1* complementation test, constructs with the FAST marker were introduced into *fd-1* and Col, and T1 seeds harboring the expression cassettes were selected from T1 seed pool by GFP fluorescence[55]. Selected T1 seeds were sterilized, sown on soil and grown under LD condition. Similarly selected Col and *fd-1* T1 plants harboring the empty vector were used as the control.

Transformation of *Nicotiana benthamiana* leaves

Agrobacterium tumefaciens strain GV3101::pMP90 harboring expression plasmids was grown in LB medium supplemented with appropriate antibiotics, harvested by centrifugation at 5,000 rpm for 10 min, and resuspended in 10 mM $MgCl_2$ and 150 μM acetosyringone. The cultures were incubated at room temperature for 2 h and infiltrated into *N. benthamiana* leaves using a 1-ml syringe. The leaf samples were harvested 2 days after infiltration for observation by confocal microscopy.

Yeast two-hybrid assay

The Matchmaker GAL4 Two-Hybrid System 3 (Clontech) was used. Protein coding regions of wild-type and mutant versions of FD, FT, and 14-3-3 were cloned into pCUY[54] or pGAD424. Yeast two-hybrid assays were performed using yeast strain AH109. The appropriate plasmids were transformed into the yeast strain by the lithium acetate method and were selected on SCD plates lacking leucine and tryptophan (–LW). After overnight culture of transformed yeasts on liquid YPD, yeast cells were harvested, washed, and transferred to the selection plates containing SCD lacking leucine,

tryptophan, and histidine (–LWH) for the histidine requirement test. These plates were incubated at 30°C and scored for yeast cell growth.

Bimolecular fluorescent complementation (BiFC) analysis and confocal microscopy,

BiFC analysis was performed as previously described[5]. *35S::YFP*^{C}*::FT* (YC-FT), wild-type and mutant versions of *35S::YFP*^{N}*::FD* (YN-FD, YN-T282A, and YN-T282E), *35S::YFP*^{N} (YN) and *35S::YFP*^{C} (YC) were introduced into *N. benthamiana* epidermal cells in the indicated combinations. Fluorescence was observed with a confocal laser scanning microscope (Olympus FV1000).

Co-localization analysis

35S::CPK33:EYFP:3HA:His and 35S::FD:mCherry constructs were co-introduced into epidermal cells of *N. benthamiana* leaf by *Agrobacterium*-mediated transient transformation. Leaf samples were harvested 2 days after infiltration for observation by confocal microscopy. Fluorescence was detected with a FV1000 confocal laser scanning microscope (Olympus) and signal intensity was analyzed by

ASW-10 software (Olympus).

RT-PCR analysis

RNA was extracted using Sepazol reagent (Nacalai tesque) and treated with RNase-free DNase (TaKaRa) according to the manufacture's instructions. Total RNA (1 μg) was reverse-transcribed in a 20 μl reaction mixture using Transcriptor (Roche). After the reaction, the mixture was diluted with 80 μl of TE buffer, and 1 μl aliquots were used for PCR in 20 μl of PCR mixture containing 2 μl of 10X Ex Taq buffer, 2 μl of 2 mM dNTPs, 2 μl of 10 μM each of primers, and 0.1 μl of Taq DNA polymerase. PCRs were performed with the following programs.

FD: 96°C 30sec, 55°C 30sec, 72°C 30sec; 35 cycles

LFY: 96°C 30sec, 55°C 30sec, 72°C 30sec; 35 cycles

AP1: 96°C 30sec, 60°C 30 sec, 72°C 30sec; 35 cycles

ACT2: 96°C 30sec, 55°C 30sec, 72°C 30sec; 25 cycles

CPK4 5' part: 96°C 30sec, 60°C 30 sec, 72°C 30sec; 30 cycles

CPK4 3' part: 96°C 30sec, 55°C 30 sec, 72°C 30sec; 30 cycles

CPK6 5’ part: 96°C 30sec, 55°C 30sec, 72°C 30sec; 30 cycles

CPK6 3’ part: 96°C 30sec, 58°C 30sec, 72°C 30sec; 30 cycles

CPK33 5’ part: 96°C 30sec, 55°C 30sec, 72°C 30sec; 30 cycles

CPK33 3’ part: 96°C 30sec, 55°C 30sec, 72°C 30sec; 30 cycles.

For amplification of the entire coding region of CPK genes, 1 μl aliquots of cDNA were used for PCR in 20 μl of PCR mixture containing 10 μl of 2X KOD FX Neo, 2 μl of 2 mM dNTPs, 2 μl of 10 μM each primers, 5% dimethyl sulfoxide, and 0.5 μl of KOD FX Neo DNA polymerase (TOYOBO). Touchdown PCR method was employed. During first cycles, annealing temperature is decreased in 0.5°C per cycle. Detail PCR program was described as follows.

For first half cycles: 96°C 30sec, 65 to 50°C 30sec, 68°C 2min; 30 cycles

For second half cycles: 96°C 30sec, 50°C 30sec, 68°C 2min; 30 cycles

Primers are listed in Table S2.

Quantitative RT-PCR analysis

Plants were grown under LD condition and harvested to collect shoot apices

after 7 days at ZT15. RNA was extracted and cDNA was synthesized as described in RT-PCR analysis. Quantitative RT-PCR analyses were performed with a CFX96 system (Bio-Rad laboratories). Two-μl aliquots were used for PCR in a 20 μl reaction mixture containing 2 μl of 10X Ex Taq buffer, 2.4 μl of 2 mM dNTPs, 0.5 μl of 10 μM each primers, 0.8 μl of 50 mM $MgSO_4$, 10X SYBR mix, and 0.2 μl of Taq DNA polymerase. Reactions were performed with the following programs.

CPK6: 95°C 10sec, 65°C 15sec, 72°C 15sec; 40 cycles

CPK33: 95°C 10sec, 60°C 15sec, 72°C 15sec; 40 cycles

FLC: 95°C 10sec, 68°C 15sec, 72°C 15sec; 40 cycles

TUB: 95°C 10sec, 60°C 15sec, 72°C 15sec; 40 cycles

Primers are listed in Table S2.

Spatial expression analysis at shoot apex

To detect EGFP and EYFP fluorescence at the shoot apex, 7-day-old transgenic plants of *pCPK6::CPK6:EYFP:3HA:His*, *pCPK33::CPK33:EYFP:3HA:His*, and *pFD::EGFP:FD; fd-1* were embedded in 5 % agar and sectioned by a vibratome.

Sections were stained with DAPI solution (1 μg/ml DAPI, 0.1 % Triton X-100 in PBS) and were observed with a confocal laser scanning microscope (Olympus FV1000). More than five independent T1 plants were examined for CPK6 and CPK33.

References

54. Suzuki, T., Sakurai, K., Ueguchi, C. & Mizuno, T. Two types of putative nuclear factors that physically interact with histidine-containing phosphotransfer (Hpt) domains, signaling mediators in His-to-Asp phosphorelay, in Arabidopsis. *Plant Cell Physiol.* **42**, 37-45 (2001).

55. Shimada, T.L., Shimada, T. & Hara-Nishimura, I. A rapid non-destructive screenable marker, FAST, for identifying transformed seeds of *Arabidopsis thaliana*. *Plant J.* **61**, 519-528 (2010).

Table S1. List of plasmid constructs used in this study.

	Construct	Insert fragment	Template	Primer Fw	Primer Rv	Vector	Cloning methods
Protein expression	FD-His	FD CDS	FD cDNA	684	3043	pET41a	Nde I/Sal I
	GST-oligoFD-His	oligo FD	ssDNA hybridization	3401	3402	pET41a	Spe I/Xho I
	GST-T276A oligo-His	oligo T276A	ssDNA hybridization	3403	3404	pET41a	Spe I/Xho I
	GST-T282A oligo-His	oligo T282A	ssDNA hybridization	3405	3406	pET41a	Spe I/Xho I
	GST-T276A, T282A oligo –His	oligo T276A, T282A	ssDNA hybridization	3407	3408	pET41a	Spe I/Xho I
	GST-L277Q oligo His	oligo L277Q	ssDNA hybridization	4214	4215	pET41a	Spe I/Xho I
	GST-tFD	truncated FD	FD cDNA	2400	2393	pET41a	Spe I/Sal I
	GSt-tT282A	truncated T282A	FD cDNA	2400	2395	pET41a	Spe I/Sal I
	GST-FD	FD CDS	FD cDNA	700	2393	pGEX6P-1	BamH I/Sal I
	GST-FDP	FDP CDS	FDP cDNA	1327	2393	pGEX6P-1	BamH I/Sal I

	Trx-His-CPK3-CA	CPK3-CA	CPK3 cDNA	5207	5814	pET32a	In-Fusion
	Trx-His-CPK4-CA	CPK4-CA	CPK4 cDNA	5209	5815	pET32a	In-Fusion
	Trx-His-CPK5-CA	CPK5-CA	CPK5 cDNA	5211	5816	pET32a	In-Fusion
	Trx-His-CPK6-CA	CPK6-CA	CPK6 cDNA	5213	5817	pET32a	In-Fusion
	Trx-His-CPK11-CA	CPK11-CA	CPK11 cDNA	5223	5818	pET32a	In-Fusion
	Trx-His-CPK12-CA	CPK12-CA	CPK12 cDNA	5225	5819	pET32a	In-Fusion
	Trx-His-CPK26-CA	CPK26-CA	CPK26 cDNA	5253	5820	pET32a	In-Fusion
	Trx-His-CPK27-CA	CPK27-CA	CPK27 cDNA	5255	5821	pET32a	In-Fusion
	Trx-His-CPK32-CA	CPK32-CA	CPK32 cDNA	5265	5822	pET32a	In-Fusion
	Trx-His-CPK33-CA	CPK33-CA	CPK33 cDNA	5267	5823	pET32a	In-Fusion
	Trx-His-CPK4	CPK4 CDS	CPK4 cDNA	5209	6156	pET32a	In-Fusion
	Trx-His-CPK6	CPK6 CDS	CPK6 cDNA	5213	6157	pET32a	In-Fusion
	Trx-His-CPK33	CPK33 CDS	CPK33 cDNA	5267	6158	pET32a	In-Fusion
Yeast two hybrid	AD-FD	FD CDS	FD cDNA	678	689	pGAD424	BamH I/Sal I
	AD-T282A	T282A CDS	FD cDNA	690	2245	pGAD424	Sma I/Sal I
	AD-T282E	T282E CDS	FD cDNA	690	2246	pGAD424	Sma I/Sal I
	AD-L277Q	L277Q CDS	FD cDNA	690	4295	pGAD424	Sma I/Sal I
	AD-LQTE	LQTE CDS	L277Q CDS	690	4296	pGAD424	Sma I/Sal I
	BD-FT	FT CDS	FT cDNA	3371	3372	pCUY	Sma I/Sal I
	BD-GRF3 Psi	GRF3 Psi CDS	GRF3 Psi cDNA	4263	4264	pCUY	BamH I/Pst I

	BD-GRF4 Phi	GRF4 Phi CDS	GRF4 Phi cDNA	4265	4011	pCUY	Sma I/BamH I
Plant transformation	35S::YN-T282E	YN-T282E	YN-FD	1483	T282E	pBI121	XbaI/Sac I
	35S::FD	FD CDS	FD cDNA	700	695	pENTR1A/pFAST-G02	Gateway
	35S::T282A	T282A CDS	FD cDNA	700	4207	pENTR1A/pFAST-G02	Gateway
	35S::T282E	T282E CDS	FD cDNA	700	4206	pENTR1A/pFAST-G02	Gateway
	35S::L277Q	L277Q CDS	FD cDNA	700	695	pENTR1A/pFAST-G02	Gateway
	35S::LQTE	LQTE CDS	L277Q CDS	700	4206	pENTR1A/pFAST-G02	Gateway
	35S::EYFP-FD	FD CDS	FD cDNA	700	695	pPZP211/35S::EYFP	BamH I/Sac II
	35S::EYFP-T282A	T282A CDS	FD cDNA	700	4207	pPZP211/35S::EYFP	BamH I/Sac II
	35S::EYFP-T282E	T282E CDS	FD cDNA	700	4206	pPZP211/35S::EYFP	BamH I/Sac II
	35S::FD-mCherry	FD CDS	FD cDNA	700	1475	pPZP211/35S::mCherry	BamH I/Sma I
	35S::CPK1:EYFP:3HA:His	CPK1 CDS	CPK1 cDNA	5203	5204	pPZP211/35S::EYFP:3HA:His	In-Fusion
	35S::CPK3:EYFP:3HA:His	CPK3 CDS	CPK3 cDNA	5207	5208	pPZP211/35S::EYFP:3HA:His	In-Fusion
	35S::CPK4:EYFP:3HA:His	CPK4 CDS	CPK4 cDNA	5209	5210	pPZP211/35S::EYFP:3HA:His	In-Fusion
	35S::CPK5:EYFP:3HA:His	CPK5 CDS	CPK5 cDNA	5211	5212	pPZP211/35S::EYFP:3HA:His	In-Fusion
	35S::CPK6:EYFP:3HA:His	CPK6 CDS	CPK6 cDNA	5213	5214	pPZP211/35S::EYFP:3HA:His	In-Fusion
	35S::CPK7:EYFP:3HA:His	CPK7 CDS	CPK7 cDNA	5215	5216	pPZP211/35S::EYFP:3HA:His	In-Fusion
	35S::CPK8:EYFP:3HA:His	CPK8 CDS	CPK8 cDNA	5217	5218	pPZP211/35S::EYFP:3HA:His	In-Fusion
	35S::CPK9:EYFP:3HA:His	CPK9 CDS	CPK9 cDNA	5219	5220	pPZP211/35S::EYFP:3HA:His	In-Fusion
	35S::CPK10:EYFP:3HA:His	CPK10 CDS	CPK10 cDNA	5221	5222	pPZP211/35S::EYFP:3HA:His	In-Fusion

	35S::CPK11:EYFP:3HA:His	CPK11 CDS	CPK11 cDNA	5223	5224	pPZP211/35S::EYFP:3HA:His	In-Fusion
	35S::CPK12:EYFP:3HA:His	CPK11 CDS	CPK12 cDNA	5225	5226	pPZP211/35S::EYFP:3HA:His	In-Fusion
	35S::CPK13:EYFP:3HA:His	CPK11 CDS	CPK13 cDNA	5227	5228	pPZP211/35S::EYFP:3HA:His	In-Fusion
	35S::CPK15:EYFP:3HA:His	CPK11 CDS	CPK15 cDNA	5231	5232	pPZP211/35S::EYFP:3HA:His	In-Fusion
	35S::CPK21:EYFP:3HA:His	CPK21 CDS	CPK21 cDNA	5223	5224	pPZP211/35S::EYFP:3HA:His	In-Fusion
	35S::CPK23:EYFP:3HA:His	CPK23 CDS	CPK23 cDNA	5245	5246	pPZP211/35S::EYFP:3HA:His	In-Fusion
	35S::CPK26:EYFP:3HA:His	CPK26 CDS	CPK26 cDNA	5253	5254	pPZP211/35S::EYFP:3HA:His	In-Fusion
	35S::CPK27:EYFP:3HA:His	CPK27 CDS	CPK27 cDNA	5255	5256	pPZP211/35S::EYFP:3HA:His	In-Fusion
	35S::CPK28:EYFP:3HA:His	CPK28 CDS	CPK28 cDNA	5257	5258	pPZP211/35S::EYFP:3HA:His	In-Fusion
	35S::CPK30:EYFP:3HA:His	CPK30 CDS	CPK30 cDNA	5261	5262	pPZP211/35S::EYFP:3HA:His	In-Fusion
	35S::CPK31:EYFP:3HA:His	CPK31 CDS	CPK31 cDNA	5263	5264	pPZP211/35S::EYFP:3HA:His	In-Fusion
	35S::CPK32:EYFP:3HA:His	CPK32 CDS	CPK32 cDNA	5265	5266	pPZP211/35S::EYFP:3HA:His	In-Fusion
	35S::CPK33:EYFP:3HA:His	CPK33 CDS	CPK33 cDNA	5267	5268	pPZP211/35S::EYFP:3HA:His	In-Fusion
	pCPK6::CPK6:EYFP:3HA:His	Promoter CPK6	Genomic DNA	6324	6325	pPZP211/35S::CPK6:EYFP:3HA:His	In-Fusion
	pCPK33::CPK33:EYFP:3HA:His	Promoter CPK33	Genomic DNA	6322	6323	pPZP211/35S::CPK33:EYFP:3HA:His	In-Fusion

Table S2. List of primers used in this study.

Purpose	DNA fragments	Name	Sequence
Protein expression	oligo FD	3401	ctagtATGGCTGCAGCAATTCAGCAACCCAAAAAGAACACACTTCAACGGTCTTCCACAGCTCCATTTC
		3402	tcgagAAATGGAGCTGTGGAAGACCGTTGAAGTGTGTTCTTTTTGGGTTGCTGAATTGCTGCAGCCATA
	oligo T276A	3403	ctagtATGGCTGCAGCAATTCAGCAACCCAAAAAGAACGCACTTCAACGGTCTTCCACAGCTCCATTTC
		3404	tcgagAAATGGAGCTGTGGAAGACCGTTGAAGTGCGTTCTTTTTGGGTTGCTGAATTGCTGCAGCCATA
	oligo T282A	3405	ctagtATGGCTGCAGCAATTCAGCAACCCAAAAAGAACACACTTCAACGGTCTTCCGCAGCTCCATTTC
		3406	tcgagAAATGGAGCTGCGGAAGACCGTTGAAGTGTGTTCTTTTTGGGTTGCTGAATTGCTGCAGCCATA
	oligo T276A, T282A	3407	ctagtATGGCTGCAGCAATTCAGCAACCCAAAAAGAACGCACTTCAACGGTCTTCCGCAGCTCCATTTC
		3408	tcgagAAATGGAGCTGCGGAAGACCGTTGAAGTGCGTTCTTTTTGGGTTGCTGAATTGCTGCAGCCATA
	oligo L277Q	4215	ctagtATGGCTGCAGCAATTCAGCAACCCAAAAAGAACACACAACAACGGTCTTCCACAGCTCCATTTC
		4216	tcgagAAATGGAGCTGTGGAAGACCGTTGTTGTGTGTTCTTTTTGGGTTGCTGAATTGCTGCAGCCATA
	tFD	2400	actagtGTTCCATCCAGTTCTTTTGG
		2393	gtcgacTCAAAATGGAGCTGTGGAAGACC
	tT282A	2400	actagtGTTCCATCCAGTTCTTTTGG
		2395	gtcgacTCAAAATGGAGCTGCGGAAGACC
	FD (pET41a)	684	catatgATGTTGTCATCAGCTAAGCATCAGA
		3043	gtcgacAAATGGAGCTGTGGAAGACCGTT
	FD (pGEX)	700	ggatccATGTTGTCATCAGCTAAGCAT

		2393	gtcgacTCAAAATGGAGCTGTGGAAGACC
	FDP (pGEX)	1327	aacccgggggatccATGTTGTCATCAGCAAAGCA
		2393	gtcgacTCAAAATGGAGCTGTGGAAGACC
	CPK3-CA	5207	GGCTGATATCGGATCCATGGGCCACAGACACAGC
		5814	CCGCAAGCTTGTCGATTACTCCCCATCTTCTCTAA
	CPK4-CA	5209	GGCTGATATCGGATCCATGGAGAAACCAAACCCTAG
		5815	CCGCAAGCTTGTCGATTAAGCATGTTCATCAACAATC
	CPK5-CA	5211	GGCTGATATCGGATCCATGGGCAATTCTTGCCGTG
		5816	CCGCAAGCTTGTCGATTAAACACCATTCTCACAGAT
	CPK6-CA	5213	GGCTGATATCGGATCCATGGGCAATTCATGTCGTG
		5817	CCGCAAGCTTGTCGATTAAACTCCATTCTCACAGAT
	CPK11-CA	5223	GGCTGATATCGGATCCATGGAGACGAAGCCAAACC
		5818	CCGCAAGCTTGTCGATTATGCTTGTTCATCGACAAT
	CPK12-CA	5225	GGCTGATATCGGATCCATGGCGAACAAACCAAGAAC
		5819	CCGCAAGCTTGTCGATTAAACCTTATCATCCACAATC
	CPK26-CA	5253	GGCTGATATCGGATCCATGCATAGAGATCTAAAGCC
		5820	CCGCAAGCTTGTCGATTAAACTCCATTTTCACAGATC
	CPK27-CA	5255	GGCTGATATCGGATCCATGGGTTGCTTCAGCAGTA
		5821	CCGCAAGCTTGTCGATTATTCTCCTTCTTTCATCCA
	CPK32-CA	5265	GGCTGATATCGGATCCATGGGTAATTGTTGCGGAAC

		5822	CCGCAAGCTTGTCGATTATGTCTTTGCATTCTGTAAC
	CPK33-CA	5267	GGCTGATATCGGATCCATGGGGAATTGCTTAGCCA
		5823	CCGCAAGCTTGTCGATTATTCTCCACCTTCTCTTAG
	CPK4	5209	GGCTGATATCGGATCCATGGAGAAACCAAACCCTAG
		6156	CCGCAAGCTTGTCGATTACTTTGGTGAATCATCAGA
	CPK6	5213	GGCTGATATCGGATCCATGGGCAATTCATGTCGTG
		6157	CCGCAAGCTTGTCGACTACACATCTCTCATGCTG
	CPK33	5267	GGCTGATATCGGATCCATGGGGAATTGCTTAGCCA
		6158	CCGCAAGCTTGTCGATTAGAACAATCTTGGTTGTTG
Yeast two hybrid	FT	3371	cccgggGATGTCTATAAATATAAGAGACCCT
		3372	gtcgacAAGTCTTCTTCCTCCGCAGCC
	FD	678	ggatccGTATGTTGTCATCAGCTAAGCATCAGA
		689	gtcgacTCAAAATGGAGCTGTGGAAGACCGTT
	T282A	690	cccgggGATGTTGTCATCAGCTAAGCATCAGA
		2245	gtcgacAAATGGAGCTGCGGAAGACCGTTGAA
	T282E	690	cccgggGATGTTGTCATCAGCTAAGCATCAGA
		2246	gtcgacAAATGGAGCTTCGGAAGACCGTTGAA
	L277Q	690	ggatccGTATGTTGTCATCAGCTAAGCATCAGA
		4295	gtcgacTCAAAATGGAGCTGTGGAAGACCGTTGTTGTGTGTTCTT
	LQTE	690	ggatccGTATGTTGTCATCAGCTAAGCATCAGA

		4296	gtcgacTCAAAATGGAGCTTCGGAAGACCGTTGTTGTGTGTTCTT
	GRF3 Psi	4263	ggatccatATGTCGACAAGGGAAGAGAAT
		4264	ctgcagCTCGGCACCATCGGGCTTTGAT
	GRF4 Phi	4265	ccgggtATGGCGGCACCACCAGCATCAT
		4011	ggatccGATCTCCTTCTGTTCTTCAGCAGGCT
Plant transformation	YN-T282E	1483	tctagaATGGTGAGCAAGGGCGAGGA
		T282E	gagctcTCAAAATGGAGCTTCGGAAGACCGTTGAA
	FD	700	aggatccATGTTGTCATCAGCTAAGCAT
		695	ccgcggTCAAAATGGAGCTGTGGAAGA
	T282A	700	aggatccATGTTGTCATCAGCTAAGCAT
		4207	ccgcggTCAAAATGGAGCTGCGGAAGACCGTTGAA
	T282E	700	aggatccATGTTGTCATCAGCTAAGCAT
		4206	ccgcggTCAAAATGGAGCTTCGGAAGACCGTTGAA
	L277Q	700	aggatccATGTTGTCATCAGCTAAGCAT
		695	ccgcggTCAAAATGGAGCTGTGGAAGA
	LQTE	700	aggatccATGTTGTCATCAGCTAAGCAT
		4206	ccgcggTCAAAATGGAGCTTCGGAAGACCGTTGAA
	3HA:His	4910	GGGGGTTAATTAACATCTTTTACCCATACGATGTTCCTGACTATGCGGGCTATCCCTATGACGTCCCGGACTATGCAG<br>GATCCTATCCATATGACGTTCCAGATTACGCTGCTCAGTGCAGCCACCACCACCACCACCACTAGGC
		4911	CTAGTGGTGGTGGTGGTGGTGGCTGCACTGAGCAGCGTAATCTGGAACGTCATATGGATAGGATCCTGCATAGTCC

			GGGACGTCATAGGGATAGCCCGCATAGTCAGGAACATCGTATGGGTAAAAGATGTTAATTAACCCCCGC
	EYFP	5140	ggatccccgggtaccgagctcATGGTGAGCAAGGGCGAGGA
		5141	aacccaccgcggtggCTTGTACAGCTCGTCCATGC
	FD	700	aggatccATGTTGTCATCAGCTAAGCAT
		1475	aacccgggAAATGGAGCTGTGGAAGACCGT
	mCherry	3467	tccccgcggATGGTGAGCAAGGGCGAGGAGGATAAC
		3468	tccccgcggTTACTTGTACAGCTCGTCCATGCCGCC
	CPK1	5203	CACGGGGGACTCTAGATGGGTAATACTTGTGTTGG
		5204	ATGAGCTCGGTACCCGAGTTTAAGAGCAATGCTAAA
	CPK3	5207	CACGGGGGACTCTAGATGGGCCACAGACACAGC
		5208	ATGAGCTCGGTACCCCATTCTGCGTCGGTTTGG
	CPK4	5209	CACGGGGGACTCTAGATGGAGAAACCAAACCCTAG
		5210	ATGAGCTCGGTACCCCTTTGGTGAATCATCAGATTT
	CPK5	5211	CACGGGGGACTCTAGATGGGCAATTCTTGCCGTG
		5212	ATGAGCTCGGTACCCCGCGTCTCTCATGCTAATG
	CPK6	5213	CACGGGGGACTCTAGATGGGCAATTCATGTCGTG
		5214	ATGAGCTCGGTACCCCACATCTCTCATGCTGATG
	CPK7	5215	CACGGGGGACTCTAGATGGGGAATTGTTGTGGCA
		5216	ATGAGCTCGGTACCCGGTCTCGCCTTCTAATTGC
	CPK8	5217	CACGGGGGACTCTAGATGGGAAATTGTTGTGCGAG

		5218	ATGAGCTCGGTACCCATTTTCGCCTTCTAATTGCAA
	CPK9	5219	CACGGGGGACTCTAGATGGGAAATTGTTTTGCCAAG
		5220	ATGAGCTCGGTACCCGAACAGCCGAGGTTGTTG
	CPK10	5221	CACGGGGGACTCTAGATGGGTAACTGTAACGCCT
		5222	ATGAGCTCGGTACCCAACAGGAACAGTTTGTCCAG
	CPK11	5223	CACGGGGGACTCTAGATGGAGACGAAGCCAAACC
		5224	ATGAGCTCGGTACCCGTCATCAGATTTTTCACCATC
	CPK12	5225	CACGGGGGACTCTAGATGGCGAACAAACCAAGAAC
		5226	ATGAGCTCGGTACCCGACATTCATAGACTCATCAG
	CPK13	5227	CACGGGGGACTCTAGATGGGAAACTGTTGCAGATC
		5228	ATGAGCTCGGTACCCTTCGTTGCCTAGGTTCAAAG
	CPK15	5231	CACGGGGGACTCTAGATGGGTTGCTTTAGCAGCA
		5232	ATGAGCTCGGTACCCTTGGACTGGAAGAATTTTCC
	CPK21	5243	CACGGGGGACTCTAGATGGGTTGCTTCAGCAGTA
		5244	ATGAGCTCGGTACCCATGGAATGGAAGCAGTTTCC
	CPK23	5245	CACGGGGGACTCTAGATGGGTTGTTTCAGCAGTAA
		5246	ATGAGCTCGGTACCCGTGGAATGGATACTGTTTCC
	CPK26	5253	CACGGGGGACTCTAGATGCATAGAGATCTAAAGCC
		5254	ATGAGCTCGGTACCCCTGCGAGACGGCATTGTT
	CPK27	5255	CACGGGGGACTCTAGATGGGTTGCTTCAGCAGTA

		5256	ATGAGCTCGGTACCCATTGATGATCGGAAGAAGTTC
	CPK28	5257	CACGGGGGACTCTAGATGGGTGTCTGTTTCTCCG
		5258	ATGAGCTCGGTACCCTCGAAGATTCCTGTGACCT
	CPK30	5261	CACGGGGGACTCTAGATGGGTAATTGTATCGCCTG
		5262	ATGAGCTCGGTACCCAACTGCAATAGATTGTCCAG
	CPK31	5263	CACGGGGGACTCTAGATGGGTTGCTACAGCAGTA
		5264	ATGAGCTCGGTACCCCTTGATCGGAAGAAGCTCC
	CPK32	5265	CACGGGGGACTCTAGATGGGTAATTGTTGCGGAAC
		5266	ATGAGCTCGGTACCCTCTTGTATCACCATTGACCT
	CPK33	5267	CACGGGGGACTCTAGATGGGGAATTGCTTAGCCA
		5268	ATGAGCTCGGTACCCGAACAATCTTGGTTGTTGTG
	pCPK6	6324	GGCCAGTGCCAAGCTTACATAAGTTGTTAAGGAGTTT
		6325	GAATTGCCCATCTAGGAAACAAACTCTCTCTATTTC
	pCPK33	6322	GGCCAGTGCCAAGCTGCGTTTCTTGAGAGGCAAG
		6323	CAATTCCCCATCTAGTGCCCGTTCGTCTTTGCT
RT-PCR	AP1	486	GCACATCCGCACTAGAAAAAA
		487	CTTCTTGATACAGACCACCCA
	FD	1527	TAATCTTCATACCCACCATCAC
		1528	CAATCCCCAAAAGAGAAACAAG
	LFY	1688	ACGCCGTCATTTGCTACTCT

		1689	CTTTCTCCGTCTCTGCTGCT
	ACT2	2151	AGAGATTCAGATGCCCAGAAGTCTTGTTCC
		2152	AACGATTCCTGGACCTGCCTCATCATACTC
	CPK4 3’ part	2717	TTATGGATGCGGCGGATATAGACA
		2718	GAGTTCTCATTTCTCAATTCCAAGT
	CPK4 5’ part	6116	GAGAAACCAAACCCTAGAAGACC
		6117	CAGGTGCAACATAATACGGAC
	CPK6 3’ part	6023	TAGATCAAGACAACGATGGACGGAT
		6024	TGTTACAAACAAACAAAACAGAGAG
	CPK6 5’ part	6118	ATTACCCAACCAGAAACAGCAACA
		6119	TACGCCGCATAATGGGTTCTTT
	CPK33 3’ part	6031	AAGATGGGAGTGGATACATTACGACA
		6032	TGTTATCTTCCTCTGTTATATACTT
	CPK33 5’ part	6120	AGGCCAGTTTGGAGTAACGTAT
		6121	ATACACTCTCCCTTCTTCGATAA
	CPK4 CDS	6116	GAGAAACCAAACCCTAGAAGACC
		2718	GAGTTCTCATTTCTCAATTCCAAGT
	CPK6 CDS	2467	ATGGGCAATTCATGTCGTGG
		2468	CTACACATCTCTCATGCTGA
	CPK33 CDS	6322	GGCCAGTGCCAAGCTGCGTTTCTTGAGAGGCAAG

		6323	CAATTCCCCATCTAGTGCCCGTTCGTCTTTGCT
qRT-PCR	CPK6	1784	AGACAACGATGGACGGATTG
		1785	CTCCCTACACCAGCATTTCC
	CPK33	1782	CGCTGATAATGACGGTAGAATC
		1783	TTCCCACTTCTCATCATAGCAC
	FLC	2816	CCGAACTCATGTTGAAGCTTGTTGAG
		2817	CGGAGATTTGTCCAGCAGGTG
	TUB	1230	CAAGCTTTCGGAGGTCAGAG
		1891	CAGCTTTGGTGATTTGAAC

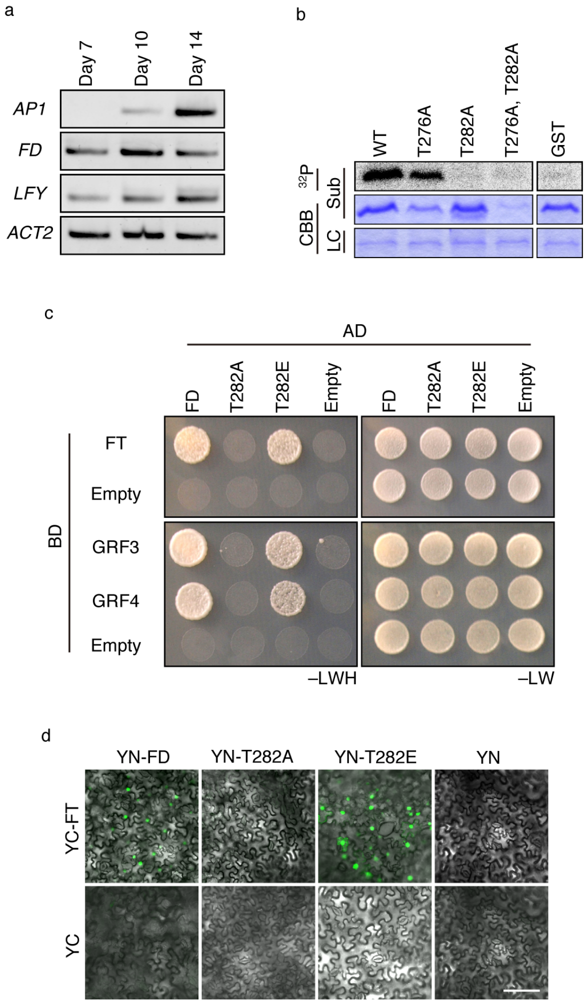

a
Day 7
Day 10
Day 14
AP1
FD
LFY
ACT2
b
WT
T276A
T282A
T276A, T282A
GST
32P
Sub
CBB
LC
c
AD
FD
T282A
T282E
Empty
BD
FT
Empty
GRF3
GRF4
–LWH
–LW
d
YN-FD
YN-T282A
YN-T282E
YN
YC-FT
YC

Supplementary Fig. S1. Phosphorylation of FD T282 and protein interaction

(**a**) Expression of *FD, AP1*, and *LEAFY* (*LFY*) in the plant samples used for kinase assays. Plants were grown under LD conditions and shoot apices were collected at Zeitgeber time 15 on Day 7, 10, and 14. *ACTIN 2* (*ACT2*) was used as a reference. RT-PCR conditions are described in *Supplementary Information*. (**b**) *In vitro* phosphorylation assay of FD T282. Wild-type (WT) and mutant versions of C4 peptide fused to GST or GST were used as a substrate. Protein extracts from shoot apices of 7-day-old plants were used as a kinases source. ^{32}P and CBB panels show autoradiography and Coomassie Brilliant Blue (CBB) staining images, respectively, of parts of the gel. Substrate (Sub) and loading control (LC, showing a band around the molecular mass of RubisCO large subunit) panels confirm that similar amounts of substrates and protein extracts, respectively, were used in the experiments. (**c**) Effect of substitution of FD T282 on interaction with FT and 14-3-3s. A non-phosphorylatable (T282A) version and a phospho-mimic (T282E) version of FD were tested for interaction with FT and two isoforms of 14-3-3, GRF3 (14-3-3 psi) and GRF4 (14-3-3 phi). –LWH and –LW indicate selective (SCD –Leu, –Trp, -His) and non-selective (SCD –Leu, –Trp) medium, respectively. (**d**) Effect of substitution of FD T282 on interaction with FT *in planta* examined by bi-molecular fluorescent complementation assay in *Nicotiana benthamiana* epidermal cells. N-terminal half of YFP (YN) fused to wild-type and mutant versions of FD (YN-FD, YN-T282A, and YN-T282E), YN, C-terminal half of YFP (YC) fused to FT (YC-FT), and YC were introduced into

epidermal cells in the indicated combinations. All images are in the same magnification.

Scale bar: 100 μm.

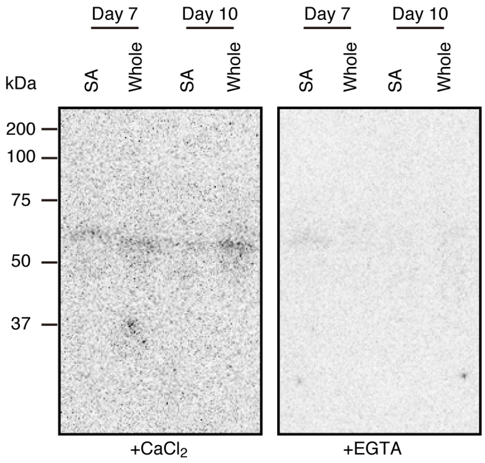

Supplementary Fig. S2. In-gel kinase assay without substrates.

Extracts from shoot apices (SA) or whole aerial parts (Whole) of plants of indicated ages were analyzed. Kinase reactions were performed in the presence (+$CaCl_2$) or absence (+EGTA) of Ca^{2+}.

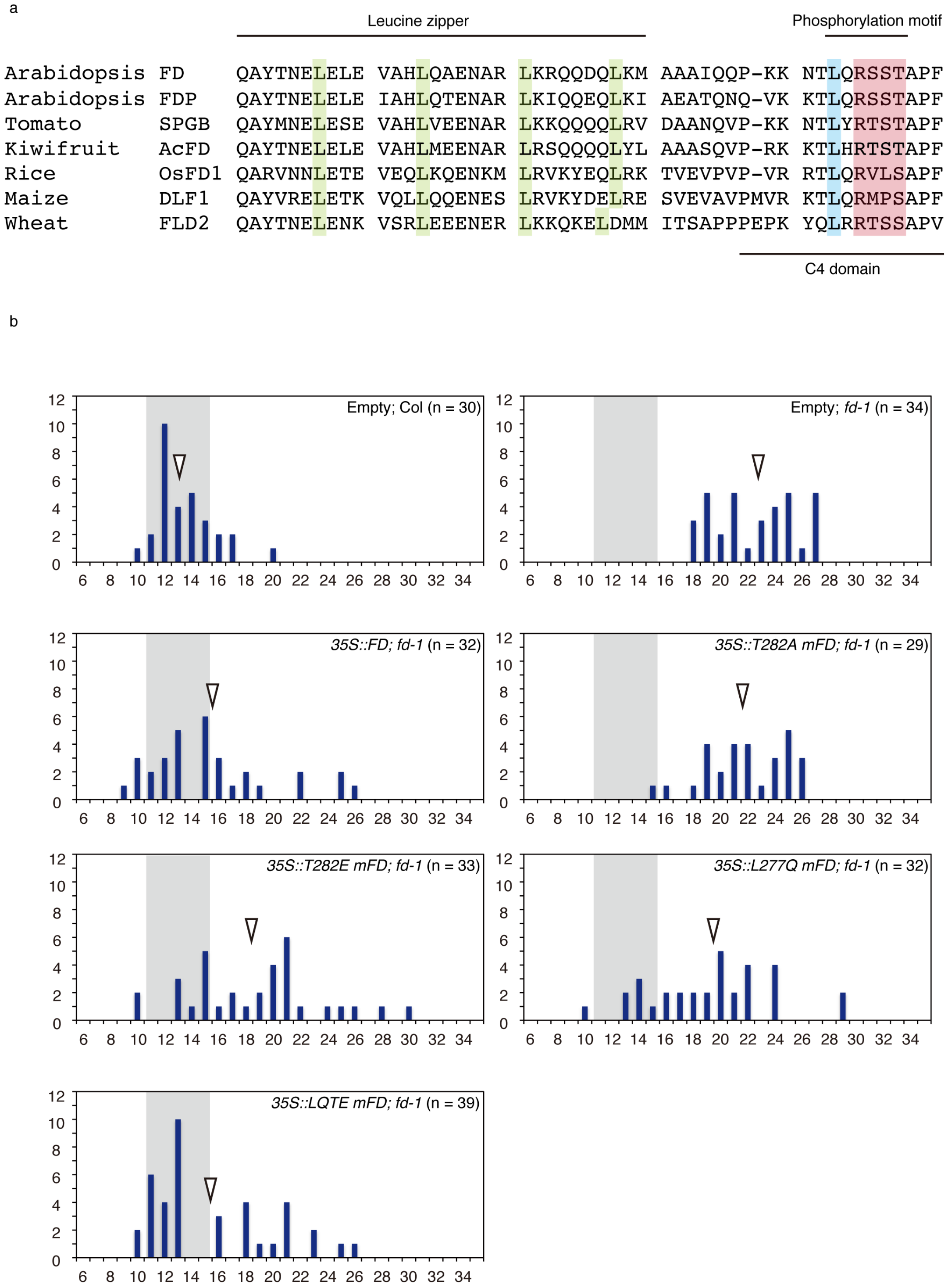
a
Leucine zipper
Phosphorylation motif
Arabidopsis FD QAYTNELELE VAHLQAENAR LKRQQDQLKM AAAIQQP-KK NTLQRSSTAPF
Arabidopsis FDP QAYTNELELE IAHLQTENAR LKIQQEQLKI AEATQNQ-VK KTLQRSSTAPF
Tomato SPGB QAYMNELESE VAHLVEENAR LKKQQQQLRV DAANQVP-KK NTLYRTSTAPF
Kiwifruit AcFD QAYTNELELE VAHLMEENAR LRSQQQQLYL AAASQVP-RK KTLHRTSTAPF
Rice OsFD1 QARVNNLETE VEQLKQENKM LRVKYEQLRK TVEVPVP-VR RTLQRVLSAPF
Maize DLF1 QAYVRELETK VQLLQQENES LRVKYDELRE SVEVAVPMVR KTLQRMPSAPF
Wheat FLD2 QAYTNELENK VSRLEEENER LKKQKELDMM ITSAPPPEPK YQLRRTSSAPV
C4 domain
b
Empty; Col (n = 30)
Empty; fd-1 (n = 34)
35S::FD; fd-1 (n = 32)
35S::T282A mFD; fd-1 (n = 29)
35S::T282E mFD; fd-1 (n = 33)
35S::L277Q mFD; fd-1 (n = 32)
35S::LQTE mFD; fd-1 (n = 39)

Supplementary Fig. S3. Effect of sequence alterations in the C4 domain on FD activity

(**a**) Sequence alignment of the C-terminal region of FD and its orthologous proteins. A crucial leucine residue for phosphorylation is highlighted by blue. Red box indicates a previously proposed core consensus sequence (R/K-X-X-S/T) for phosphorylation by CDPK/SnRK protein kinase family. Leucine residues in the leucine zipper are highlighted by pale green. (**b**) Effect of sequence alterations in the C4 domain on FD activity to complement *fd-1*. Wild-type and mutant versions of FD (T282A mFD, T282E mFD, L277Q mFD, and LQTE mFD) under the control of CaMV *35S* RNA (*35S*) promoter were introduced into *fd-1*. Wild-type Col and *fd-1* plants with an empty vector were used as control transgenic plants. Distribution of flowering time (measured by the number of rosette leaves, horizontal axis) in T1 plants under LD conditions is shown for each transgenic genotype. Vertical axis represents the number of plants. Shaded area in each graph indicates the range of rosette leaf number of Empty; Col transgenic plants (average ± 1 standard deviation). Vertical open arrowheads indicate the average of rosette leaf number for each genotype.

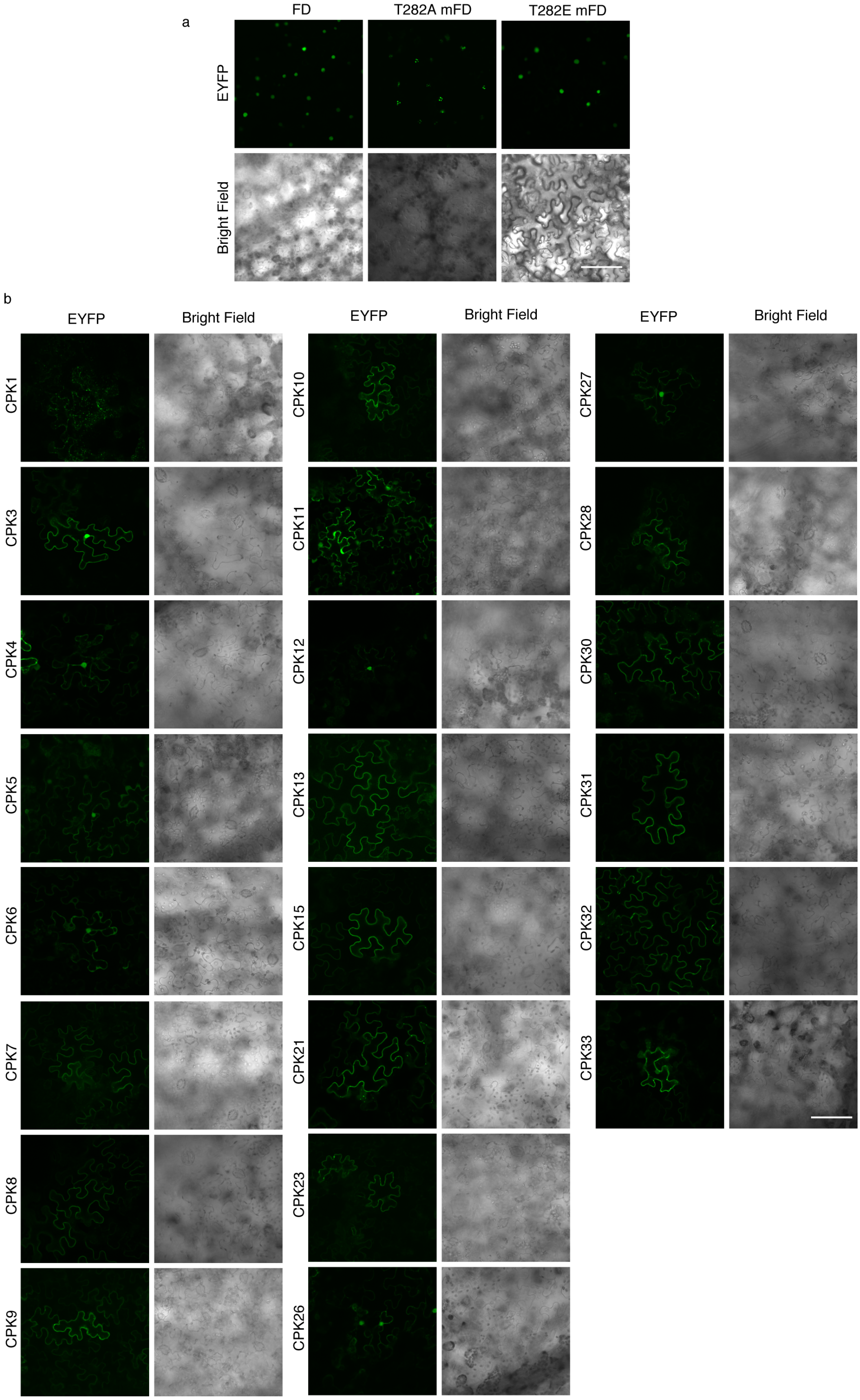

a
FD
T282A mFD
T282E mFD
EYFP
Bright Field
b
EYFP
Bright Field
EYFP
Bright Field
EYFP
Bright Field
CPK1
CPK3
CPK4
CPK5
CPK6
CPK7
CPK8
CPK9
CPK10
CPK11
CPK12
CPK13
CPK15
CPK21
CPK23
CPK26
CPK27
CPK28
CPK30
CPK31
CPK32
CPK33

Supplementary Fig. S4. Subcellular localization of FD and CDPKs.

(**a**) Subcellular localization of wild-type and C4 domain mutant versions of FD transiently expressed in *Nicotiana benthamiana* leaf epidermal cells. (**b**) Subcellular localization of CDPK transiently expressed in *N. benthamiana* leaf epidermal cells. All the CPK proteins were expressed as a fusion with EYFP. EYFP fluorescence and bright-field images are shown. All images are in the same magnification. Scale bar: 100 μm.

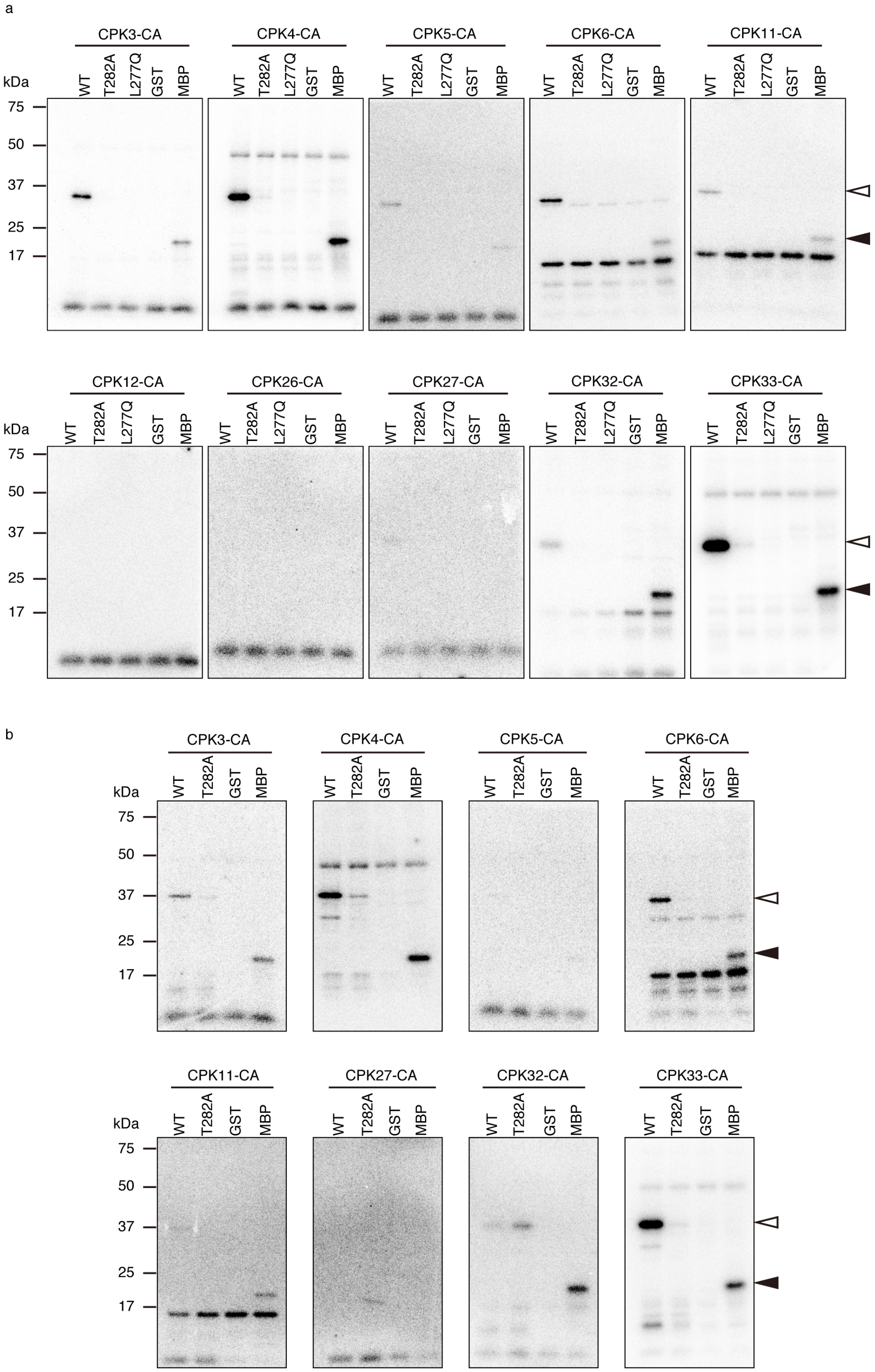
a
CPK3-CA
CPK4-CA
CPK5-CA
CPK6-CA
CPK11-CA
WT
T282A
L277Q
GST
MBP
kDa
75
50
37
25
17
CPK12-CA
CPK26-CA
CPK27-CA
CPK32-CA
CPK33-CA
b
CPK3-CA
CPK4-CA
CPK5-CA
CPK6-CA
CPK11-CA
CPK27-CA
CPK32-CA
CPK33-CA

Supplementary Fig. S5. *In vitro* phosphorylation of FD T282 by CPK-CAs.

(**a**) Wild-type and mutant versions of C4 peptide fused to GST were used as substrates. White and black arrowheads indicate phosphorylation signals from GST-tagged C4 peptide and MBP, respectively. Panels for CPK4-CA, CPK6-CA, and CPK33-CA are uncropped images of the autoradiograms shown in Fig. 4b. (**b**) tFD and its mutant derivatives fused to GST were used as substrates. White and black arrowheads indicate phosphorylation signals from GST-tagged tFD and MBP, respectively. In both experiments, GST and MBP were used as controls. CPK-CAs were constructed based on published information[38] and primers used for cloning are shown in Table S2.

a

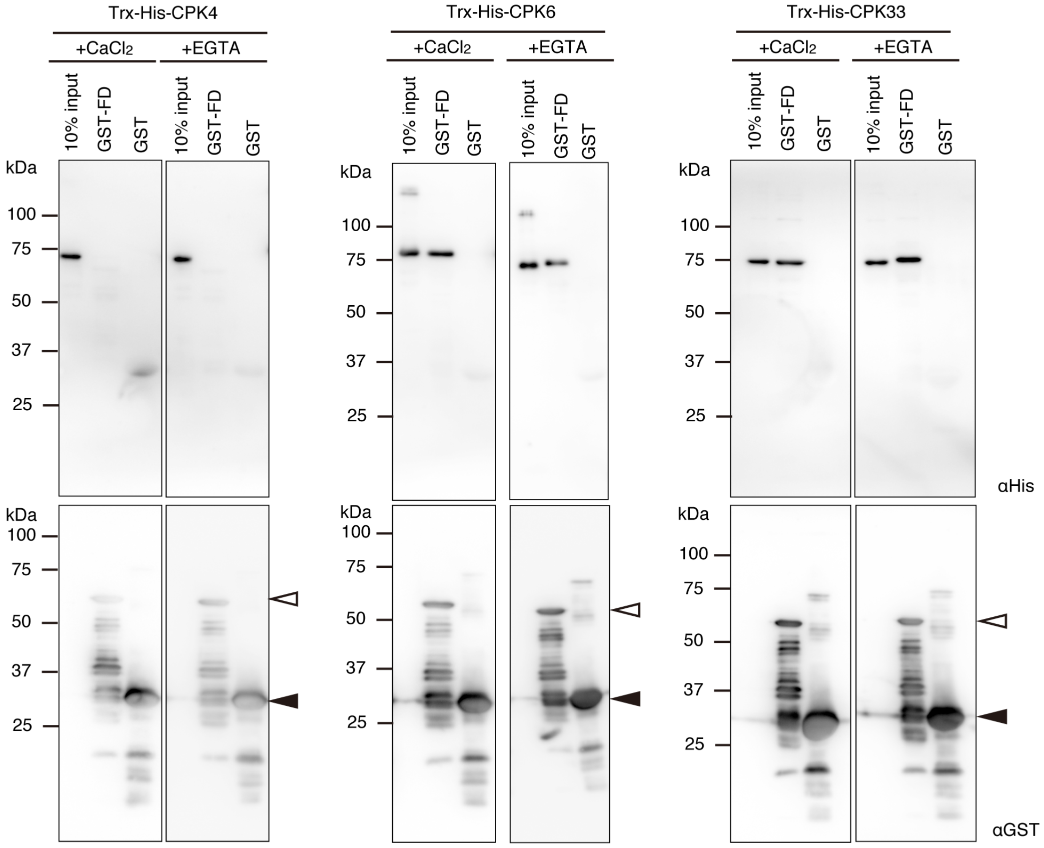
Trx-His-CPK4
+CaCl2
+EGTA
10% input
GST-FD
GST
kDa
100
75
50
37
25
Trx-His-CPK6
Trx-His-CPK33
αHis
αGST

b

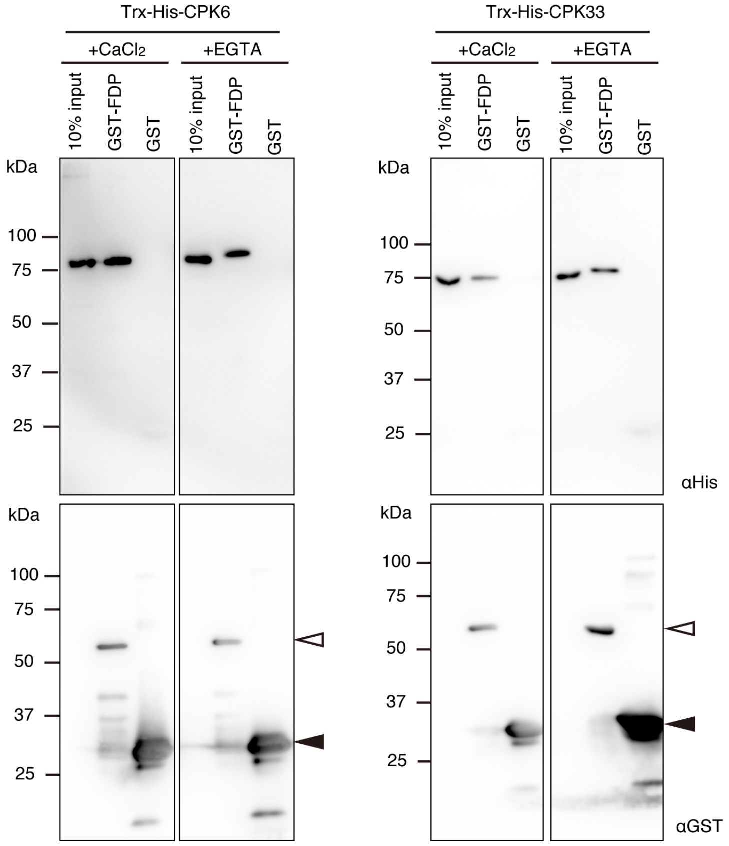
Trx-His-CPK6
+CaCl2
+EGTA
10% input
GST-FDP
GST
kDa
100
75
50
37
25
Trx-His-CPK33
αHis
αGST

Supplementary Fig. S6. Interaction of FD and FDP with CPK6 and CPK33 *in vitro*

(**a**) Uncropped images of the immunoblots shown in Fig. 4c (with anti-His tag antibody, αHis) and immunoblots with anti-GST antibody (αGST). One-tenth volumes of the reactions were loaded in "10 % input" lanes. Black and white arrowheads indicate positions of GST-FD and GST, respectively. (**b**) Uncropped images of the immunoblots shown in Fig. 4d (with anti-His tag antibody, αHis) and immunoblots with anti-GST antibody (αGST). Trx-His-CPKs were pulled-down with either GST-FDP or GST in the presence (+$CaCl_2$) or absence (+EGTA) of Ca^{2+}. One-tenth volumes of the reactions were loaded in "10 % input" lanes. Black and white arrowheads indicate positions of GST-FDP and GST, respectively.

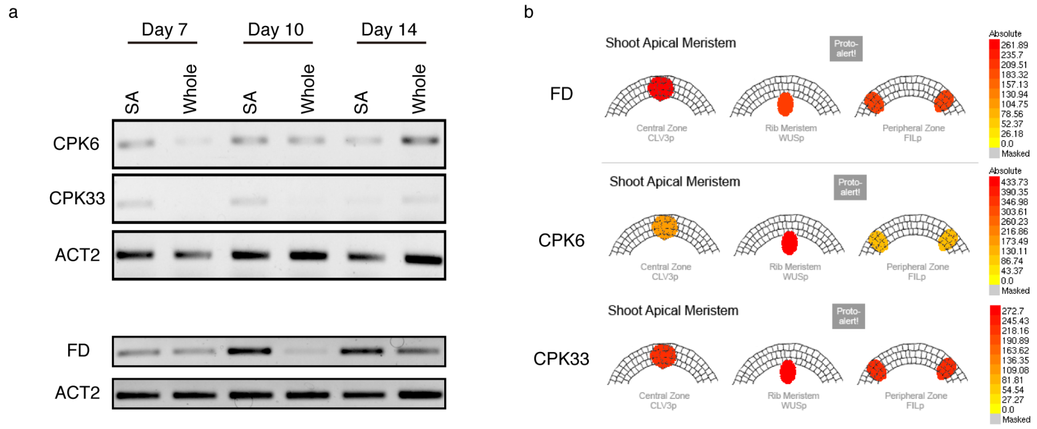

Supplementary Fig. S7. Expression profiles of *FD*, *CPK6*, and *CPK33*.

(**a**) RT-PCR analysis of FD, CPK6, and CPK33 expression in shoot apices (SA) and whole aerial parts (Whole) from plants of the indicated ages grown in LD conditions. RT-PCR conditions are described in *Supplementary Information*. *ACT2* was used as a reference. (**b**) Spatial expression pattern of FD, CPK6, and CPK33 in shoot apex. Images were retrieved from an Arabidopsis eFP browser (http://bar.utoronto.ca/efp/cgi-bin/efpWeb.cgi).

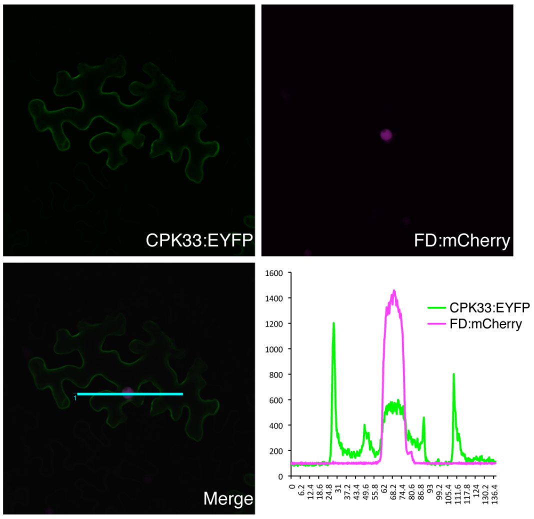

Supplementary Fig. S8. Co-localization of CPK33 and FD proteins in the nucleus.

CPK33 and FD proteins were co-expressed in *N. benthamiana* leaf epidermal cells as a fusion protein with either EYFP (CPK33) or mCherry (FD). Fluorescence images were obtained by confocal laser scanning microscopy. Fluorescence intensity along the indicated line (from left to right) on the mergned image was quantified by ASW-10 software (Olympus). Vertical axis and horizontal axis in the graph indicate fluorescence intensity and the position on line (distance (µm) from the left end), respectively.

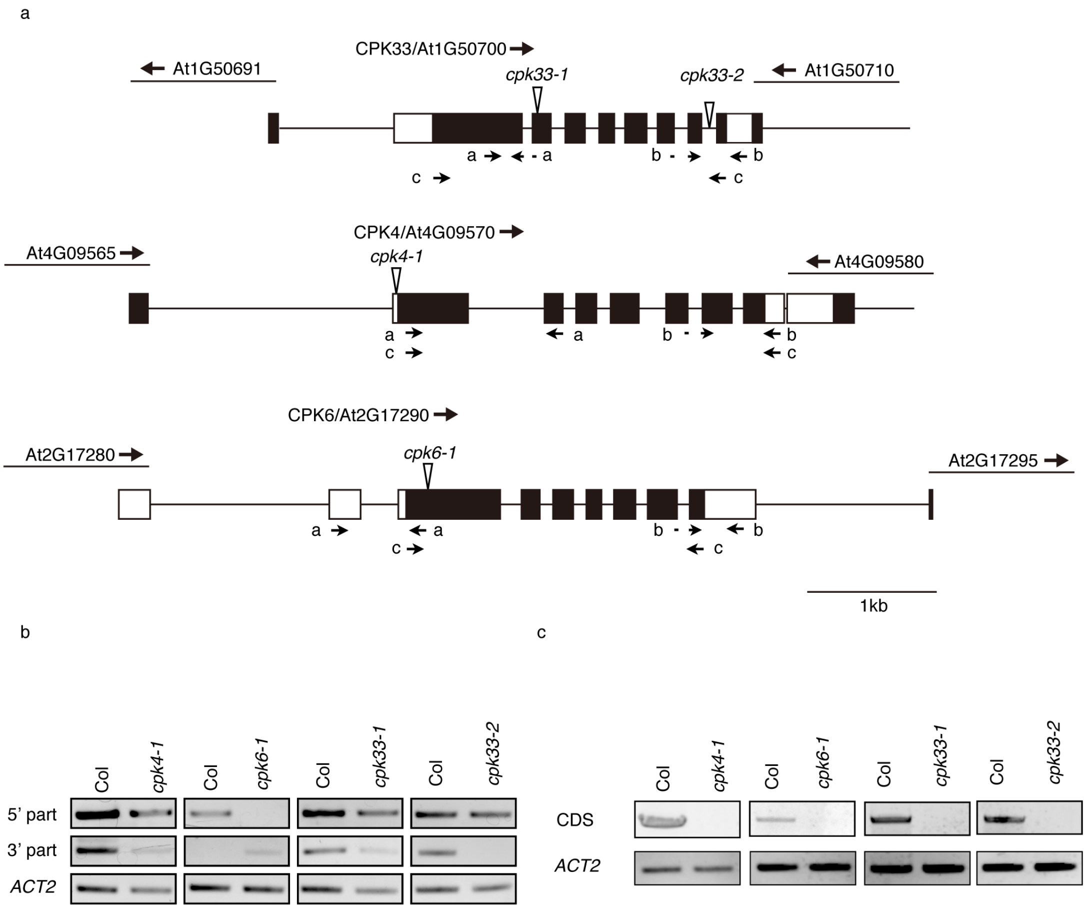
a
CPK33/At1G50700
At1G50691
cpk33-1
cpk33-2
At1G50710
a
a
b
b
c
c
CPK4/At4G09570
At4G09565
cpk4-1
At4G09580
a
a
b
b
c
c
CPK6/At2G17290
At2G17280
cpk6-1
At2G17295
a
a
b
b
c
c
1kb
b
Col
cpk4-1
Col
cpk6-1
Col
cpk33-1
Col
cpk33-2
5’ part
3’ part
ACT2
c
Col
cpk4-1
Col
cpk6-1
Col
cpk33-1
Col
cpk33-2
CDS
ACT2

Supplementary Fig. S9. *cpk4*, *cpk6*, and *cpk33* T-DNA insertion mutants.

(**a**) Genomic regions of *CPK4*, *CPK6*, and *CPK33*. White and Black boxes indicate untranslated regions and coding regions, respectively. Inverted triangles indicate the positions of a T-DNA insertion. Small solid and broken arrows indicate primer positions used for RT-PCR analysis. For each gene, primers were designed to amplify 5' and 3' parts (a and b, respectively) and the entire coding region (c) of the transcript. T-DNA is inserted in the coding region of kinase domain in *cpk6-1* and *cpk33-1*. (**b**), (**c**) Analysis of CPK transcripts in the T-DNA insertion mutants by RT-PCR. The 5' and 3' parts (**b**) and the entire coding region (**c**) were amplified. RT-PCR conditions are described in *Supplementary Information*. Primer sequences are shown in Table S2. *ACT2* was used as a reference.

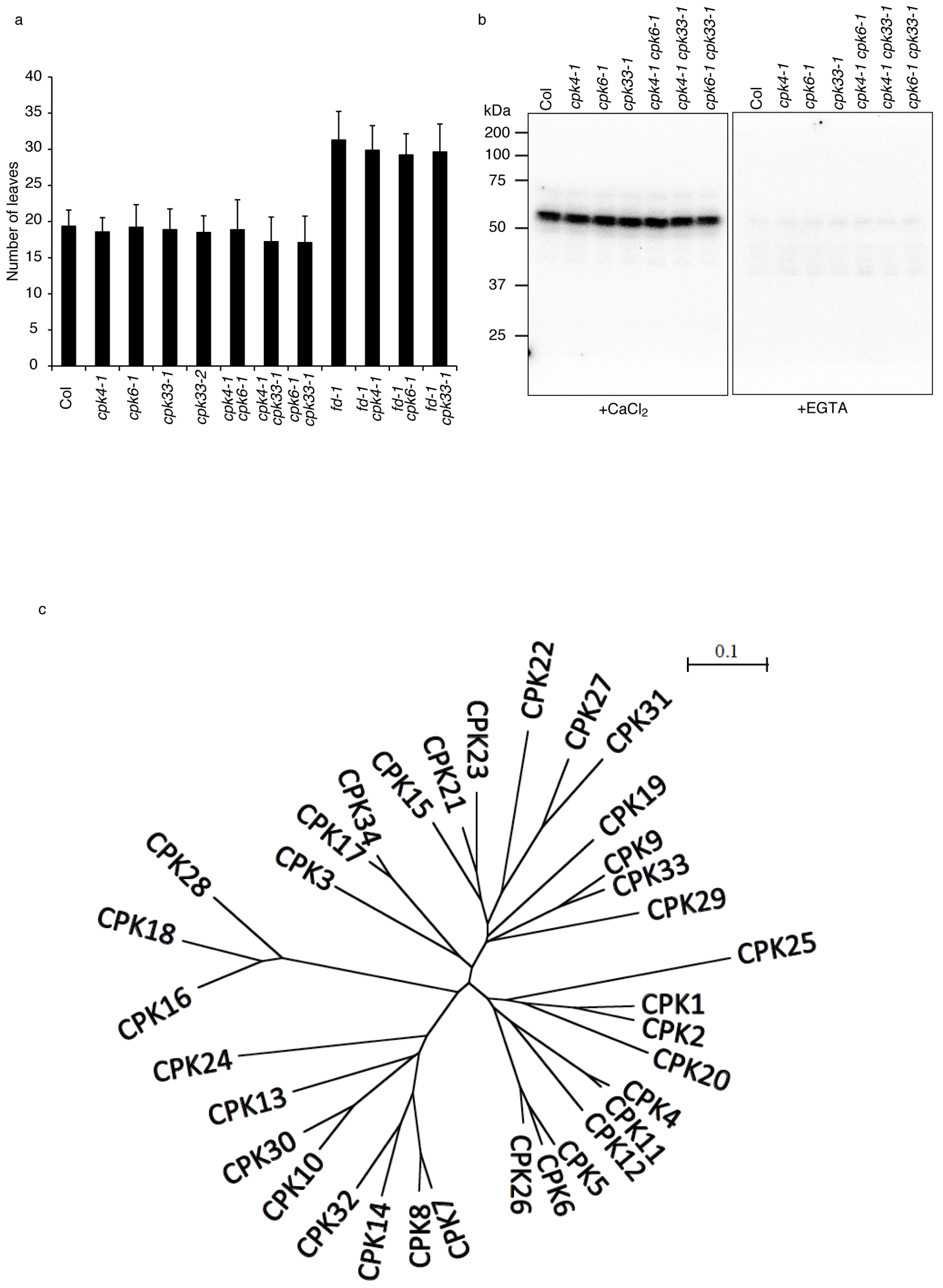
a
Number of leaves
0
5
10
15
20
25
30
35
40
Col
cpk4-1
cpk6-1
cpk33-1
cpk33-2
cpk4-1 cpk6-1
cpk4-1 cpk33-1
cpk6-1 cpk33-1
fd-1
fd-1 cpk4-1
fd-1 cpk6-1
fd-1 cpk33-1
b
kDa
200
100
75
50
37
25
Col
cpk4-1
cpk6-1
cpk33-1
cpk4-1 cpk6-1
cpk4-1 cpk33-1
cpk6-1 cpk33-1
+CaCl2
+EGTA
c
0.1
CPK22
CPK27
CPK31
CPK23
CPK21
CPK15
CPK34
CPK17
CPK3
CPK19
CPK9
CPK33
CPK29
CPK28
CPK18
CPK16
CPK25
CPK1
CPK2
CPK20
CPK4
CPK11
CPK12
CPK5
CPK6
CPK26
CPK24
CPK13
CPK30
CPK10
CPK32
CPK14
CPK8
CPK7

Supplementary Fig. S10. Effects of *cpk* mutations on flowering and FD phosphorylation.

(**a**) Flowering time of *cpk* single and double mutants and *cpk fd* double mutants in LD conditions. The number of leaves at flowering (mean ± SD) is shown (n = 16 for *fd-1 cpk33-1*, n = 21-24 for other genotypes). (**b**) Phosphorylation of FD T282 by the in-gel kinase assay with protein extracts from shoot apices of *cpk* single and double mutants as a kinase source. (**c**) A unrooted phylogenetic tree of CDPK proteins in Arabidopsis.

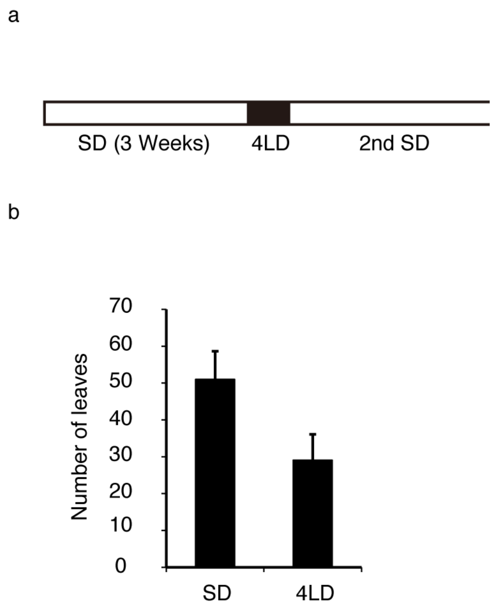

Supplementary Fig. S11. Four LDs inserted into SD induces flowering.

(a) Growth regime for SD/4LD/SD condition. Plants were grown under SD condition for 3 weeks, subjected to 4 days of LD condition, and then transferred back to the SD condition. (b) Wild-type plants (Col) were grown under SD condition (SD, n = 19) and SD/4LD/SD condition (4LD, n = 16), respectively. The number of rosette and cauline leaves was counted after flowering (mean ± SD). There is a statistically significant difference ($P < 0.001$, Student's t-test [two-tailed test]).
